# Localised states and their capture characteristics in amorphous phase-change materials

**DOI:** 10.1038/s41598-019-43035-7

**Published:** 2019-04-29

**Authors:** Martin Rütten, Andreas Geilen, Abu Sebastian, Daniel Krebs, Martin Salinga

**Affiliations:** 1 I. Physikalisches Institut (IA), RWTH Aachen University, Sommerfeldstrasse 14, 52074 Aachen, Germany; 2 IBM Research - Zurich, Säumerstrasse 4, 8803 Rüschlikon, Switzerland

**Keywords:** Electronic properties and materials, Electronic devices, Information storage, Electronic properties and materials, Optical spectroscopy

## Abstract

As phase-change materials are poised to play a key role in next-generation computing systems, improving the current understanding of electrical transport in their amorphous phase can further strengthen their technological competitiveness. Even though the interaction of charge carriers with disorder-induced localised states largely affect the field-dependent conductivity, a clear link between electrical transport and specific features of the electronic density of states (DOS) could not be established yet due to a lack of knowledge of the capture characteristics of trap states. Here, we address this knowledge gap and employ modulated photocurrent spectroscopy (MPC) to investigate localised states in the frequently studied amorphous phase of Ge_2_Sb_2_Te_5_. Additionally, we present results on the DOS in the bandgap of amorphous AgIn-doped Sb_2_Te, which has not been subject to high-resolution DOS spectroscopy before. We find experimental evidence for clearly non-constant capture coefficients among a continuous spectrum of localised states in both studied materials. According to this observation especially in AgIn-doped Sb_2_Te, where no pronounced defect can be detected as main channel for carrier emission, we point out the necessity of modifying the current Poole-Frenkel-based transport modelling.

## Introduction

Driven by the explosive growth of data and the speed-gap between the processing unit and conventional storage systems, memory industry introduced the storage class memory (SCM) as next generation, non volatile memory class, satisfying key requirements such as speed, reliability and efficiency at a minimum cost-per-bit ratio^1,2^. The most promising SCM candidate to reshape the memory landscape is phase-change memory (PCRAM)^3,4^ as a representative of resistively switching memories, which has been shown to be technologically mature and commercially viable since the introduction of 3D-XPoint^TM^ by Intel and Micron Technology in 2015^5^. In 2014, the large potential of PCRAM as SCM was attested, when a prototype PCI-e card including PCRAM chips combined with flash memory and DRAM provided 275 times higher speed compared to a traditional SSD^6^. PCRAM is also finding applications in emerging non-von Neumann computing paradigms such as brain-inspired neuromorphic computing^7,8^.

PCRAM encodes logical information by means of the electrical resistance of the memory device, utilising that so-called phase-change materials (PCM) can be switched reversibly between a high-resistive amorphous (reset) and a low-resistive crystalline (set) state. Resetting a PCRAM cell is commonly obtained by melt-quenching a portion of the previously crystalline PCM, while the set state results from bringing the amorphous PCM up to a temperature regime of high atomic mobility and thus fast crystallisation^9^. Switching to the set state is decisively facilitated by the so-called threshold switching event, which occurs as breakdown of electrical resistivity at elevated electrical fields and allows for sufficient Joule heating in the otherwise low-conductive amorphous phase. Preceding the threshold switch, a highly non-linear current-voltage characteristic can be observed, which plays a key role in switching PCRAM devices fast and energy-efficiently^10^. Even though subthreshold electrical conduction in the semiconducting amorphous phase has been extensively studied so far, the underlying mechanism of its non-linear field dependence is still debated^11,12^.

Already in the 1970s^13,14^, field-dependent conductivity in amorphous GeTe was attempted to be understood by investigating the interaction between charge carriers and localised states in the bandgap of amorphous semiconductors^15^. In 2007, Ielmini and Zhang^16^ proposed a widely recognised model for electrical transport in melt-quenched amorphous Ge_2_Sb_2_Te_5_ PCRAM devices by invoking the Poole-Frenkel (PF) emission of trapped charge carriers^17–21^. However, the Ielmini-Zhang-model of charge carriers hopping with a constant travelling distance between neighbouring states is incompatible with the well-known band transport concept^11^. Le Gallo *et al*.^22^ addressed this shortcoming by proposing a PF-based transport model that describes field-dependent conductivity in amorphous Ge_2_Sb_2_Te_5_ and GeTe remarkably well without assuming immediate re-trapping of emitted carriers. Nonetheless, PF-based transport modelling so far only includes trap states attributed to a single energy level, which is commonly attributed to a structural defect at a specific energetic distance to the band edge^16,22–24^. This approach appears plausible at least for PCM containing germanium such as amorphous GeTe, where under- or over-coordinated Ge-atoms have been associated with electronic defect states in the bandgap^25–27^. However, in germanium-free PCM, which might lack an appropriate defect as main channel for PF assisted charge carrier emission, the applicability of current PF-based transport modelling is questionable. This presumption is strengthened by the fact that previous PF-based transport modelling on amorphous Ag_4_In_3_Sb_67_Te_26_ yields an unreasonably large intertrap distance^12^.

Recently, Kaes and Salinga made clear by linking subthreshold electrical transport and the density of electronic states (DOS) that more precise knowledge on the density of localised states, but also on capture characteristics of such states is needed to better understand electrical transport in amorphous PCM^24^. The capture characteristic of a localised state with respect to a certain type of charge carrier is typically expressed by the capture coefficient *c* = *σ* · *υ*_th_ as product of thermal velocity *υ*_th_ and capture cross section *σ*^28,29^. As such, *c* measures the efficacy of a localised state for capturing a charge carrier. In p-type conductivity materials such as amorphous PCM^30^, the capture coefficient for holes *c*_p_ is of predominant importance for electrical transport.

A key technique to obtain experimental input on both density and capture coefficient of localised states in the bandgap of amorphous materials is modulated photocurrent spectroscopy (abbreviated MPC, sometimes also referred to as IMPS for intensity-modulated photocurrent spectroscopy). Decisively shaped by works of Brüggemann, Longeaud and others^29,31^, MPC spectroscopy has been used for numerous investigations of the DOS in a large variety of different materials, from amorphous silicon to lead sulfide quantum dot arrays^32–36^. Other methods to examine the electronic structure of amorphous PCM films such as x-ray or ultraviolet photoemission spectroscopy typically do not offer a sufficient energy resolution to observe distinct features of the bandgap^37–39^. The working principle of MPC spectroscopy is based on intensity-modulated illumination with photons, which generates free charge carriers in the material leading to a modulated photocurrent. During the experiment, the photocurrent amplitude and phase shift with regard to the sinusoidally modulated illumination are recorded as function of modulation frequency. These experimental data can be linked to the density of localised states by interpreting the measured amplitude and phase shift in terms of free charge carriers that are trapped by localised states and released back to the band after some delay. In particular, it has been commonly agreed upon that the phase shift between excitation and modulated photocurrent predominantly originates from the thermal emission of trapped charge carriers (e.g.^31,40,41^). In this picture, photons are solely generating free charge carriers, they are not expected to contribute to the measured MPC signal in any other way, e.g. via optically induced transitions involving localised states.

So far, MPC spectroscopy was used in studies by Longeaud *et al*.^42^ and Luckas *et al*.^43,44^ to gain remarkable insight into the DOS of amorphous GeTe and Ge_2_Sb_2_Te_5_. According to their MPC studies, the bandgap of these PCM exhibits Gaussian-shaped peaks of localised states related to structural defects. In addition to these pronounced peaks, disorder-induced spectra of localised states were found with exponentially increasing density towards both band edges. Especially with respect to these spectra of localised states, up to now the analysis of MPC results has been performed under the restrictive assumption that the investigated states exhibit one common capture coefficient (*c*_p_ = *const*.)^42–44^. This simplification makes it much easier to analytically link experimental MPC data to the DOS, since *c*_p_ may then be assumed to be independent of energy^29^.

In the present work, we challenge this assumption of a constant capture coefficient based on precise experimental input for the density and capture characteristics of localised states. We suggest crucial improvements to the already established method of MPC spectroscopy, including both experimental and analysis-related aspects. Our study focuses on high-quality MPC measurement of two materials. Amorphous Ge_2_Sb_2_Te_5_ represents the probably most popular and widely studied PCRAM material^45^. By additionally choosing amorphous AgIn-doped Sb_2_Te (more precisely Ag_4_In_3_Sb_67_Te_26_), we provide very first insights into the DOS in the bandgap of this germanium-free PCM that stands out due to its drift characteristics, a property relevant for storing multiple levels in a single PCRAM element^46,47^.

## Results

### MPC spectroscopy measurements

**Figure 1:**
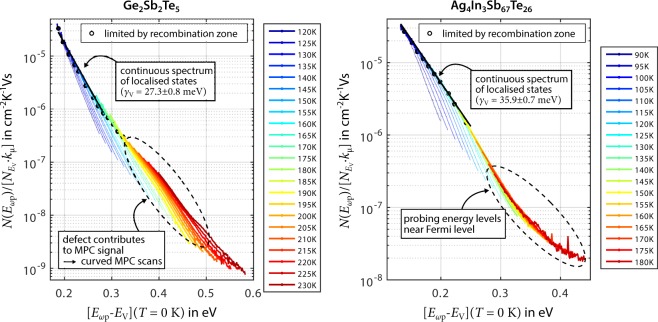
MPC spectroscopy data sets assuming homogeneous capture characteristics on amorphous Ge_2_Sb_2_Te_5_ and Ag_4_In_3_Sb_67_Te_26_. MPC scans recorded at various temperatures are plotted as MPC DOS (see equation 4) against energy (with valence band edge *E*_V_ as reference, see equation 6), based on the assumption of *c*_p_ = const. for involved states. Data points that are identified as unaffected by the recombination zone are plotted bold, and the limits are marked by circles. The thin lines of the same color going beyond those circles represent data affected by the recombination zone, which are therefore ignored in the further analysis. Preliminarily scaling MPC scans with one common capture coefficient for holes (${k}_{{\rm{c}}}{N}_{{E}_{{\rm{V}}}}=5\cdot {10}^{13}\,{{\rm{s}}}^{-{\rm{1}}}\,{{\rm{K}}}^{-1/2}\,{{\rm{eV}}}^{-{\rm{1}}}$) yields a somewhat coherent MPC DOS in both materials. A continuous spectrum of localised states is observed, for which the MPC DOS appears to decrease exponentially from the valence band edge towards the bandgap center (see local fits). A slight curvature in MPC scans for Ge_2_Sb_2_Te_5_ at elevated temperatures reveals the effect of a structural defect located at a specific energetic distance from the band edge. MPC scans for Ag_4_In_3_Sb_67_Te_26_ appear to be straight except from a flattening at elevated temperatures (*T* ≥ 150 K), which is most likely an artefact due to probing energy levels close to the Fermi level.

Figure 1 presents the obtained MPC spectroscopy measurements on amorphous Ge_2_Sb_2_Te_5_ and Ag_4_In_3_Sb_67_Te_26_. Due to the experimentally demonstrated p-type conductivity of amorphous PCM^30^, the MPC signal is most likely dominated by trap and release processes of holes^29,42^. These holes interact with localised states within the bandgap at energy levels below the Fermi level, which is located in the lower half of the bandgap near the bandgap center. Contributions to the MPC signal from electrons interacting with localised states above the Fermi level can be neglected. This implies that only localised states below the Fermi level can be probed by MPC spectroscopy. Apart from this, it is a common constraint in MPC analysis that exact values for quantities such as the DOS at the band edge $N({E}_{{\rm{V}}})={N}_{{E}_{{\rm{V}}}}$ or the extended-state mobility *μ*_ext,p_ are not known. Consequently, instead of plotting the pure DOS *N* as function of probed energy *E*_ωp_, usually a so-called *MPC DOS* being proportional to *N* is plotted against energy (see methods).

Looking at Fig. 1, it can be seen that a coherent DOS is composed of locally overlapping MPC scans, which are recorded at different temperatures. Each MPC scan originates from measuring amplitude and phase shift of the photocurrent while sweeping through the modulation frequency. During this process, the modulation frequency determines at which energy level localised states are probed. More precisely, using a higher modulation frequency implies that localised states at an energy level closer to the valence band edge are probed. Eventually, the essential analysis step is to find the correct capture coefficient for holes *c*_p_ of the probed localised states, because only then the aggregation of individual MPC scans yields a coherent DOS. Considering that *c*_p_ itself depends on temperature *T* as in *c*_p_(*T*) = *k*_c_ · *T*^1/2^ due to the thermal velocity of charge carriers^29^, we suggest to introduce the factor *k*_c_. Thereby, the product ${k}_{{\rm{c}}}{N}_{{E}_{{\rm{V}}}}$ can be used as temperature-independent tuning parameter for the matching process of MPC scans (see methods).

During this process of determining the capture characteristics of localised states, it is crucial to consider that charge carriers trapped at energy levels closer to the bandgap center (and thus measured at lower modulation frequencies) have an increasing probability of recombining instead of being re-emitted to the band. Since the MPC analysis employed in the present work can only handle an MPC signal dominated by trap- and release processes, segments of MPC scans corresponding to energy levels dominated by recombination traffic must not be used to form a coherent MPC DOS and are therefore excluded from further analysis (marked as thin lines in Fig. 1). For identifying which segments must be excluded, we employ an experimental procedure in which MPC scans for a specific temperature are recorded at various light fluxes (see methods).

At first sight, for both materials MPC scans seem to form a somewhat coherent MPC DOS as constant capture characteristics (${k}_{{\rm{c}}}{N}_{{E}_{{\rm{V}}}}=5\cdot {10}^{13}\,{{\rm{s}}}^{-{\rm{1}}}\,{{\rm{K}}}^{-1/2}\,{{\rm{eV}}}^{-{\rm{1}}}$) are assumed for the probed localised states in the bandgap (Fig. 1). A continuous spectrum of localised states is observed, for which the MPC DOS appears to decrease exponentially from the valence band edge towards the bandgap center. Local exponential fits reveal slopes (also called decay energies *γ*_V_) of 27.3 ± 0.8 meV (Ge_2_Sb_2_Te_5_) and 35.9 ± 0.7 meV (Ag_4_In_3_Sb_67_Te_26_) close to values previously found in MPC spectroscopy studies on amorphous PCM^24,42^.

As Ag_4_In_3_Sb_67_Te_26_ has a higher conductivity compared to Ge_2_Sb_2_Te_5_, it is possible to measure MPC scans with satisfactory signal-to-noise ratio for Ag_4_In_3_Sb_67_Te_26_ down to 90 K, while low-temperature data on Ge_2_Sb_2_Te_5_ are limited to 120 K. With regard to our results for Ge_2_Sb_2_Te_5_, MPC scans recorded at temperatures around 200 K and corresponding to energy levels closer to the bandgap center seem to be separated from the continuous spectrum of localised states. These separated MPC scans are characterised by poorer overlap and a slight, but explicit curvature. This observation fits with studies by Luckas *et al*.^43,44,48^, revealing that a pronounced peak of localised states could be probed in amorphous Ge_2_Sb_2_Te_5_ attributed to a structural defect at a specific energetic distance to the band edge. To still achieve a coherent DOS, the authors had to choose clearly different capture characteristics for the peak compared to the continuous spectrum of localised states. Observing two features in the DOS with considerably different capture characteristics means that the MPC signal is a superposition of two different MPC signals. If the different contributions to such an ambiguous MPC signal are not separated correctly, the analysis yields falsified results for the MPC DOS and corresponding capture coefficients. To avoid such a misinterpretation of experimental data in the following, MPC scans attributed to the continuous spectrum of localised states are evaluated separately from those MPC scans that show signs of a curvature.

### Continuous spectrum of localised states

When compared to existing MPC studies^36,44,49,50^, the results from Fig. 1 might already provide an acceptable overlap of MPC scans attributed to the continuous spectrum of localised states. However, as it turned out during the analysis of both materials, the value of ${k}_{{\rm{c}}}{N}_{{E}_{{\rm{V}}}}$ ensuring an optimally coherent MPC DOS seems to vary along the spectrum by at least one order of magnitude. This might already indicate that the assumption of a constant capture coefficient cannot be maintained. Still, the process applied so far of visually judging whether the single MPC scans overlap and form a coherent DOS appears to be strikingly imprecise for properly challenging the assumption of *c*_p_ = const. Thus, we objectified this procedure to achieve a more quantitative MPC analysis procedure, wherein the MPC DOS is divided into numerous slices (*bins*). A precise value for ${k}_{{\rm{c}}}{N}_{{E}_{{\rm{V}}}}$ is then calculated locally for each bin by an algorithm programmed in *MATLAB*. According to this algorithm, MPC DOS data points that are recorded at different temperatures and found in the same MPC DOS bin are scaled to meet at a common energy level (see methods).

**Figure 2:**
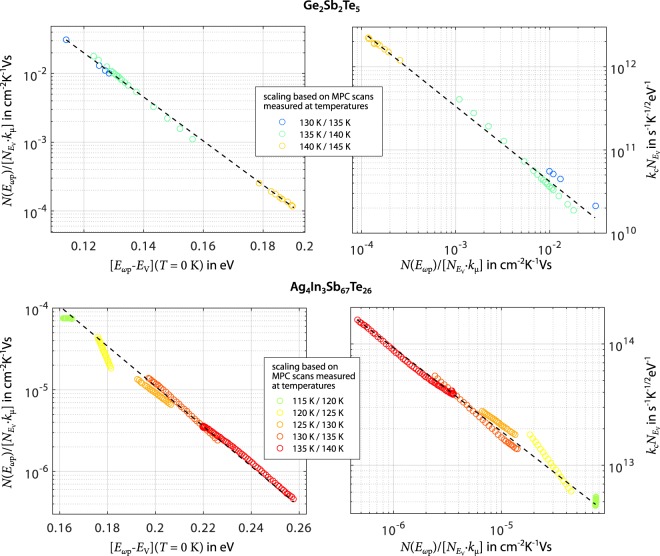
Results of the proposed quantitative MPC analysis for challenging the assumption of *c*_p_ = const. Based on the mathematical condition to ensure overlap among neighbouring MPC scans, MPC DOS data for the continuous spectrum of localised states are scaled to common energy levels if they are found in the same MPC DOS bin. This allows for a quantitative and local determination of the product ${k}_{{\rm{c}}}{N}_{{E}_{{\rm{V}}}}$. In both materials, the resulting MPC DOS exponentially decreases towards the bandgap center, with lower energy levels probed at lower temperatures (see left panels). Most strikingly, a significant gradient in ${k}_{{\rm{c}}}{N}_{{E}_{{\rm{V}}}}$ is observed. States closer to the band edge are characterised by lower capture coefficients for holes, revealed by an increase of ${k}_{{\rm{c}}}{N}_{{E}_{{\rm{V}}}}$ by more than one order of magnitude towards the bandgap center (see right panels). It is noteworthy that MPC data points in both panels follow a coherent behaviour, regardless of the temperature they are measured at. Compared to Fig. 1, not the full temperature range covered by experimental data can be used for scaling. At lower temperatures, this is due to insufficient overlap between scans at different temperature for the matching procedure. At higher temperatures, the measured MPC signal is distorted by additional contributions from the defect (in the case of Ge_2_Sb_2_Te_5_) or states near the Fermi level (in the case of Ag_4_In_3_Sb_67_Te_26_) and thus is excluded to avoid misinterpretation of the experimental data.

Figure 2 shows the result of the proposed quantitative MPC analysis for challenging the assumption of *c*_p_ = const. Since no MPC scans are excluded due to the appearance of a defect in the case of Ag_4_In_3_Sb_67_Te_26_, the amount of MPC DOS data being scaled by overlapping is considerably larger compared to the Ge_2_Sb_2_Te_5_ data set. Apart from this, in both materials the MPC DOS of the continuous spectrum of localised states is still found to decrease exponentially towards the bandgap center (see left panels of Fig. 2). More importantly, the product ${k}_{{\rm{c}}}{N}_{{E}_{{\rm{V}}}}$ increases by more than one order of magnitude when moving from the band edge towards the bandgap center (see right panels of Fig. 2). This gradient in ${k}_{{\rm{c}}}{N}_{{E}_{{\rm{V}}}}$ does not appear to be a matter of temperature as all MPC data points follow a coherent behaviour, regardless of the temperature they were measured at. Since ${N}_{{E}_{{\rm{V}}}}$ marks the DOS at the band edge and therefore is a constant reference point for all localised states, the increase in ${k}_{{\rm{c}}}{N}_{{E}_{{\rm{V}}}}$ towards the bandgap center must arise from an increase in the capture coefficient for holes *c*_p_(*T*) = *k*_c_ · *T*^1/2^ of the probed localised states. This striking observation quantitatively confirms the above mentioned indication for a non-constant capture coefficient along the continuous spectrum of localised states.

This result clearly conflicts with existing MPC spectroscopy work on amorphous Ge_2_Sb_2_Te_5_ and in general amorphous PCM^42–44^, which has always been restricted to the assumption of *c*_p_ = const. and in which MPC data has given no reason to hypothesise locally varying capture coefficients. Compared to existing studies (e.g.^36,44,49,50^), the newly introduced quantitative matching algorithm in our study benefits from the high signal-to-noise ratio, large data point density in the frequency domain and small temperature step size between MPC scans. Moreover, the present work proves the necessity to include a flux-dependent analysis of the MPC signal as preceeding step, which is not given for previous studies.

With respect to the compelling experimental evidence for non-constant capture characteristics in the continuous spectrum of localised states, it has to be noted that the currently applied MPC analysis still relies on a framework in which *c*_p_ is treated as energy-independent constant^29^. Since this assumption turns out to be invalid in the present case, the results shown in Fig. 2 must not be used to extract any concrete DOS characteristics. For now, the only conclusion regarding the continuous spectrum of localised states that can be drawn based on our MPC spectroscopy results is that the capture coefficient is non-constant.

In view of this outcome, extracting concrete DOS characteristics for amorphous PCM from MPC spectroscopy data would require that the true functional dependence of *c*_p_ for the continuous spectrum of localised states would be included in the analysis. This task could be approached by identifying an underlying mechanism of charge carrier trapping and emission that has been absent in MPC studies so far and that implies inhomogeneous capture characteristics. The involvement of multiple phonons in the transitions to and from localised states as described by Mott and Davis is a potential candidate for such a mechanism^51^. According to related studies by Baranovskii *et al*. on glassy semiconductors^52–54^, the multiphonon nature of charge carrier transitions between bands and localised states causes the probability for such transitions to decrease exponentially with increasing energy distance. This exponential energy dependence could be considered as additional factor in the capture coefficient *c*_p_. In principle, the current MPC analysis framework could be extended towards such an exponential energy dependence of *c*_p_, but establishing an analytical link between the DOS and the experimental MPC data becomes less straightforward (see section S6 in Supplement). Eventually, a revised MPC analysis starting out from the present work might turn out to require the application of numerical methods.

### Localised states attributed to a structural defect

**Figure 3:**
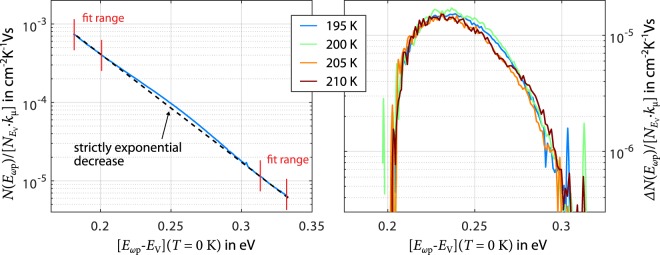
Scaling of MPC scans associated with a structural defect in amorphous Ge_2_Sb_2_Te_5_. MPC scans recorded for amorphous Ge_2_Sb_2_Te_5_ at temperatures around 200 K exhibit a curvature presumably due to a structural defect at a specific energetic distance to the band edge. Outer parts of the MPC scans seem to follow a strictly exponential decrease indicating that some contribution by the localised states of the continuous spectrum is still present in these MPC scans (example for *T* = 195 K shown in the left panel). Subtracting the exponential decrease effectively amplifies the curvature and thus helps to determine ${k}_{{\rm{c}}}{N}_{{E}_{{\rm{V}}}}=2\cdot {10}^{10}\,{{\rm{s}}}^{-{\rm{1}}}\,{{\rm{K}}}^{-1/2}\,{{\rm{eV}}}^{-{\rm{1}}}$ resulting in satisfying overlap of MPC scans (right panel).

As mentioned above, MPC scans recorded for amorphous Ge_2_Sb_2_Te_5_ at temperatures around 200 K exhibit a curvature presumably due to a structural defect at a specific energetic distance to the band edge. Recent computations show that under- or over-coordinated Ge-atoms could constitute the majority of defects in the bandgap of amorphous GeTe^25–27^. In this light, it seems plausible that we find said curvature only in our MPC scans for Ge_2_Sb_2_Te_5_ and not for Ag_4_In_3_Sb_67_Te_26_ as germanium-free PCM. Regarding the observed curved shape, it is noticeable that MPC scans presented by Longeaud *et al*. on one of the defects in GeTe^42^ are characterised by a bell-shaped peak, while MPC scans in the present work only exhibit a slight curvature. Inspecting the four MPC scans at *T* = 195 K − 210 K (see Fig. 3) reveals that the outer parts of the MPC scans seem to follow a strictly exponential decrease, which suggests that some contribution by the localised states of the continuous spectrum is still present in these MPC scans. Subtracting the exponential decrease amplifies the curvature and thus helps to determine a suitable value for ${k}_{{\rm{c}}}{N}_{{E}_{{\rm{V}}}}$ resulting into satisfying overlap of MPC scans. As a result, the MPC scans from *T* = 195 K − 210 K are scaled with ${k}_{{\rm{c}}}{N}_{{E}_{{\rm{V}}}}=2\cdot {10}^{10}\,{{\rm{s}}}^{-{\rm{1}}}\,{{\rm{K}}}^{-1/2}\,{{\rm{eV}}}^{-{\rm{1}}}$, leading to a peak position at approximately 0.25 eV. This energetic position relative to the band edge agrees well with previous MPC spectroscopy studies^43,44,48,55^. However, the above identified necessity to revise the current MPC analysis framework means that also absolute numbers derived for the defect in amorphous Ge_2_Sb_2_Te_5_ regarding position and capture coefficient must be treated with caution.

## Discussion

Even though a revision of the current MPC analysis seems necessary before quantitative DOS characteristics can be extracted from our MPC results, we can already report two significant findings regarding the DOS of amorphous PCM. These are a) the observation of a non-constant capture coefficient for a spectrum of localised states in the bandgap of both studied materials and b) the apparent lack of a structural defect at a specific energetic distance to the band edge below the Fermi level in amorphous Ag_4_In_3_Sb_67_Te_26_. These two findings are particularly relevant for subthreshold electrical transport. Current PF-based transport models for amorphous PCM only include trap states at a single energy level. For well-known PCMs like GeTe or Ge_2_Sb_2_Te_5_, it has been possible to both observe such localised electronic states (for example by MPC spectroscopy^42,43^) and attribute them to structural defects involving germanium atoms^25,26^. In the scenario of germanium-free Ag_4_In_3_Sb_67_Te_26_ apparently lacking such a singular defect as main channel for charge carrier emission, a realistic electrical transport model would need to account for trap- and release processes of charge carriers with a continuous spectrum of localised states. Such a revised transport model would also have to consider our finding of non-constant capture coefficients among the continuous spectrum of localised states, which indicates that some states in the bandgap interact more strongly with charge carriers than others. These conclusions on subthreshold electrical transport in amorphous Ag_4_In_3_Sb_67_Te_26_ should also be of importance for the technologically highly relevant threshold switching in amorphous PCM. While popular threshold switching models so far rely on trap centers at a single energy level^56,57^, it appears likely in view of the above that not only subthreshold conduction, but also threshold switching in Ag_4_In_3_Sb_67_Te_26_ demands a different explanation accounting for the presence of a continuous spectrum of localised states. Ultimately, a transport model that is able to explain the typical current-voltage characteristics of amorphous PCM as a consequence of a continuous spectrum of localised states within the bandgap should be applicable also to PCMs like GeSbTe and might thus render the existence of sharp defect levels less relevant for the observed electrical properties.

## Methods

### Experimental

Lateral devices were fabricated for MPC spectroscopy measurements, with 100 nm thick PCM material (Ag_4_In_3_Sb_67_Te_26_ or Ge_2_Sb_2_Te_5_) deposited on a substrate in between two tungsten electrodes. To prevent the fully as-deposited amorphous PCM from oxidising, it was capped *in-situ* with a 10 nm layer of (ZnS)_80_:(SiO2)_20_. We recorded experimental data by homogeneously illuminating a complete electrically contacted sample of amorphous PCM. No further areal segmentations of the sample were applied. The active volume of amorphous PCM between the two electrodes (100 nm by 1.2 mm by 40 *μm*) is large compared to potential spatial fluctuations in the density of localised states. Hence, the measurements are of intrinsically averaging character.

Electrical measurements were conducted in a Janis ST-500-2UHT cryogenic probing station, evacuated to pressures *p* ≤ 1 · 10^−4^ mbar. While a Keithley 2400 source meter is used to apply a low-field bias voltage, the device current is amplified and converted to a voltage signal by means of a Femto DHPCA-100 transimpendance amplifier. The third essential electronic component of the setup is a HP 3562 A signal analyser, which also serves as signal generator. The modulated, monochromatic light passes through an optical attenuator (type DD-600 by OZ-optics) and is fed into the cryostat chamber (further details are listed in the Supplement Section S1). While existing MPC spectroscopy studies commonly use a Lock-in amplifier to compare excitation signal and MPC signal (see e.g.^41^), the HP 3562 A performs a single-point FFT analysis of the input signal, offering a remarkable measurement speed when measuring the amplitude of the modulated photocurrent |*I*_ac_| and its phase shift *ϕ* with respect to the excitation signal. This allows for conducting the flux-dependent MPC spectroscopy described below in a time-efficient manner.

Before starting the MPC measurement, the optimum fibre position for a most homogeneous sample illumination was determined following an automated procedure. Subsequently, the signal analyser performed a frequency sweep (MPC scan) for the modulation frequency *ω* from 10 Hz to 40 kHz, recording the amplitude |*I*_ac_|(*ω*) and phase shift *ϕ*(*ω*) of the modulated photocurrent at various light fluxes (see below) at a specific temperature. This procedure was repeated for various temperatures (in 5 K steps), as long as a sufficient signal-to-noise ratio was encountered. Photoconductivity in amorphous PCM commonly exhibits a peak at around 200 K (AgIn-doped Sb_2_Te) and 250 K (Ge_2_Sb_2_Te_5_), respectively^58,59^. MPC scans for temperatures above or below this peak suffer from decreasing signal-to-noise ratio, additionally impaired by exponential increasing device resistance for decreasing temperature.

### Flux-dependent MPC spectroscopy

As described in the original MPC analysis based on trap and release processes by Longeaud *et al*.^29^, an analytical expression to relate |*I*_ac_|(*ω*) and *ϕ*(*ω*) to the density of localised states can only be obtained if the probed energy levels are unaffected by recombination. Following work of Taylor and Simmons (e.g.^28^) and also Shockley and Read^60^ on occupation statistics in the non-equilibrium steady state, increasing light flux widens the so-called recombination zone around the bandgap center. We therefore propose an experimental procedure to identify, which light flux should be used to record data for |*I*_ac_|(*ω*) and *ϕ*(*ω*) with as large as possible signal-to-noise ratio and unaffected by recombination.

Recording a first MPC scan with the highest available light flux implies that probably all probed energy levels are affected by recombination due to a very large recombination zone. Repeatedly recording MPC scans at decreasing flux shrinks the recombination zone and leads to more and more energy levels (starting with levels closer to the band edge, which are probed at higher excitation frequencies) being completely unaffected by recombination. Eventually, the MPC output is independent of further lowering the flux, indicating which particular MPC scan segment can be used for further MPC analysis. Note that the explicit assignment between excitation frequency *ω* and probed energy level *E*_ωp_ is only made in the subsequent analysis as described below. Further details on this measurement procedure and exemplary raw data for flux-dependent MPC scans can be found in the Supplement Section S2.

### Analysis for DOS spectroscopy

Two essential expressions, which have been developed by Longeaud *et al*.^29^ and were used also recently for MPC DOS spectroscopy on amorphous PCM^42,43^, serve as point of departure for our DOS spectroscopy analysis:

1 $$\frac{N({E}_{{\rm{\omega }}{\rm{p}}})\cdot {c}_{{\rm{p}}}}{{\mu }_{\mathrm{ext},{\rm{p}}}}=\frac{2}{\pi {k}_{{\rm{B}}}T}\cdot \frac{q\xi S{G}_{{\rm{ac}}}\,\sin (\varphi )}{|{I}_{{\rm{ac}}}|}$$

2 $${E}_{\omega {\rm{p}}}-{E}_{{\rm{V}}}={k}_{{\rm{B}}}T\,{\rm{l}}{\rm{n}}({\nu }_{{\rm{p}}}/\omega ).$$

Equation 1 relates the recorded MPC signal (|*I*_ac_|(*ω*) and *ϕ*(*ω*)) to the DOS *N* at the probed energy level *E*_ωp_, with *c*_p_ denoting the capture coefficient for holes of the probed states, *μ*_ext,p_ the extended-state mobility of holes, *q* the elementary charge, *ξ* the applied electrical field, *k*_B_ the Boltzmann constant, *S* the current cross section, *G*_ac_ the modulated component of the photogeneration rate and *T* the sample temperature during the MPC scan. It is important to note that the MPC signal in equation 1 only relates to the amplitude and phase shift of the modulated photocurrent, while it remains unaffected by the darkcurrent running through the sample under test. Free carriers contributing to the darkcurrent do not follow the excitation signal of the illumination and therefore do not contribute to the MPC signal.

The term on the left side of equation 1 is termed MPC DOS and obtained by gathering all known quantities, i.e. parameters defining the experimental conditions and measured data, on the right side. Both quantities, the MPC DOS and the actual DOS, are proportional to each other under the assumption that all involved states have the same capture coefficient. As it is described in^29^, this assumption of *c*_p_ = const. is also essential to linking DOS and experimental MPC data in equations 1 and 2. Taking into account effects of the inhomogeneous photogeneration rate in the sample along the penetration depth^61^, the product *S* · *G*_ac_ can be substituted by *l* · *F*_ac_ with *l* denoting the electrode length and *F*_ac_ the modulated component of the light flux.

Equation 2 relates the excitation frequency *ω* to the probed energy level with respect to the valence band edge *E*_V_, originating from the fact that these states are probed, whose emission rate of trapped carriers is equal to the excitation frequency^29^. The attempt-to-escape frequency for holes *ν*_p_ is related to the capture coefficient *c*_p_ and the effective density of states at the valence band edge *N*_V,eff_ via *ν*_p_ = *c*_p_ · *N*_V,eff_. In amorphous semiconductors the latter is commonly assumed as ${N}_{V,\text{eff}}={k}_{{\rm{B}}}T\cdot {N}_{{E}_{{\rm{V}}}}$^42,62^, ${N}_{{E}_{{\rm{V}}}}$ being the DOS at the valence band edge *E*_V_. Eventually, it is noted that equations 1 and 2 are based on assuming that band transport is the dominating electrical transport mechanism for photocarriers^29^. This assumption appears to be justified for modulated photoconductivity data recorded for the present work (see Supplement Section S5).

Due to the logarithmic frequency dependence in equation 2, only a narrow part of the MPC DOS can be probed at a constant temperature, since the range of the modulation frequency is typically limited to three orders of magnitude. However, the range of probed energy levels can be extended by shifting the scanned energy levels through a variation of the sample temperature. A coherent picture of the MPC DOS can then be composed from the MPC scans for various temperatures by matching overlap regions, in which the same energy levels are probed at different temperatures. This matching is achieved by tuning *ν*_p_. The value for *ν*_p_ most suitable for matching the data then carries information about the capture characteristics (more precisely the capture coefficient *c*_p_ via *ν*_p_) of the probed states (see e.g.^29,41,43^). Hence, the proportionality constant linking the actual DOS *N*(*E*_ωp_) to the MPC DOS on the left side of equation 1 is directly affected by this matching procedure.

For the present study, we propose some modifications of equations 1 and 2. In order to obtain a quantity from MPC spectroscopy that is linked more directly to the actual DOS *N*, we bring *c*_p_ to the right side of equation 1. This step of eliminating the capture coefficient from the MPC DOS allows us to demand that MPC data points found in the same MPC DOS bin meet at a common energy level, regardless of the fact that the assumption of *c*_p_ = *const*. might not be valid. As *c*_p_ is linked to *ν*_p_ used as matching parameter in equation 2, it makes sense to operate with the same quantity in both equations: *ν*_p_ = *c*_p_ · *N*_V,eff_. Thus equation 1 is not only divided by *c*_p_, but also by *N*_V,eff_ resulting in *ν*_p_ in the denominator of the right hand side of the equation.

3 $$\frac{N({E}_{{\rm{\omega }}{\rm{p}}})}{{N}_{V,\mathrm{eff}}(T)\cdot {\mu }_{\mathrm{ext},{\rm{p}}}(T)}=\frac{1}{{c}_{{\rm{p}}}(T){N}_{V,\mathrm{eff}}(T)}\cdot \frac{2}{\pi {k}_{{\rm{B}}}T}\cdot \frac{q\xi S{G}_{{\rm{ac}}}\,\sin (\varphi )}{|{I}_{{\rm{ac}}}|}.$$

We also take into account that various quantities have implicit temperature dependencies, which is problematic for the collective analysis of MPC scans measured at various temperatures. Besides the already mentioned ${N}_{V,\text{eff}}={k}_{{\rm{B}}}T\cdot {N}_{{E}_{{\rm{V}}}}$, also the mobility *μ*_ext,p_ and the capture coefficient *c*_p_ are expected to have a certain temperature dependence: *μ*_ext,p_ = *k*_*μ*_ · 1/*T* (due to phonon scattering, see e.g.^62^) and *c*_p_ = *k*_c_ · *T*^1/2^ (due to the thermal velocity and assuming temperature-independent capture cross sections), with *k*_*μ*_ and *k*_c_ constant in T.

4 $$\frac{N({E}_{{\rm{\omega }}{\rm{p}}})}{{N}_{{E}_{{\rm{V}}}}\cdot {k}_{\mu }}=\frac{1}{{k}_{{\rm{c}}}{N}_{{E}_{{\rm{V}}}}}\cdot \frac{2}{\pi {k}_{{\rm{B}}}{T}^{\mathrm{5/2}}}\cdot \frac{q\xi l{F}_{{\rm{ac}}}\,\sin (\varphi )}{|{I}_{{\rm{ac}}}|}.$$

While the temperature dependencies on the left side cancel each other out, all temperature terms on the right can be combined in a single *T*^5/2^ term. Also, the whole equation is multiplied by the Boltzmann constant *k*_B_, which was introduced through *N*_V,eff_ before. Our modified MPC DOS (left side of equation 4) is now temperature-independent and directly proportional to the actual DOS *N*(*E*_ωp_) normalised by the DOS at the valence band edge ${N}_{{E}_{{\rm{V}}}}$. The constant *k*_*μ*_, which is treated as unknown in the present case and might be determined by Hall measurements^62^, remains as the constant of proportionality between this normalised actual DOS and the derived MPC DOS.

As the parameter *ν*_p_ used for matching MPC scans from different temperatures has been replaced by ${\nu }_{{\rm{p}}}={c}_{{\rm{p}}}\cdot {N}_{V,\mathrm{eff}}=({k}_{{\rm{c}}}{T}^{\mathrm{1/2}})\cdot ({k}_{{\rm{B}}}T{N}_{{E}_{{\rm{V}}}})$, we are now able to vary only the remaining unknowns without the temperature dependencies. Hence, while before *ν*_p_ was varied to match overlapping scans, now the product ${k}_{{\rm{c}}}{N}_{{E}_{{\rm{V}}}}$ acts as temperature-independent tuning parameter for the matching process of MPC scans. As a consequence of making the connection between *c*_p_ and *ν*_p_ explicit and dividing equation 1 by *c*_p_, the matching parameter is no longer hidden within the MPC DOS, but instead appears together with all other quantities derived from the MPC measurements on the right side of equation 4. *k*_*μ*_, in contrast, cannot be derived from the MPC data and therefore remains within the MPC DOS (on the left side of equation 4). The matching parameter ${k}_{{\rm{c}}}{N}_{{E}_{{\rm{V}}}}$ has a direct effect on both quantities, the MPC DOS (equation 4) and the energy scale (equation 5):

5 $$[{E}_{\omega {\rm{p}}}-{E}_{{\rm{V}}}](T)=r(T)\cdot [{E}_{\omega {\rm{p}}}-{E}_{{\rm{V}}}](T=0\,{\rm{K}})={k}_{{\rm{B}}}T\,\mathrm{ln}([{k}_{{\rm{c}}}{N}_{{E}_{{\rm{V}}}}{k}_{{\rm{B}}}{T}^{3/2}]/\omega ).$$

Here, we indicated also that the energetic distance between probed energy level *E*_ωp_ and valence band edge *E*_V_ can be temperature-dependent. In light of Varshni’s widely recognised work^63^ on the temperature-dependence of the bandgap *E*_G_(*T*) = *E*_G_(*T* = 0 K) − *δ*(*T*) with *δ*(*T*) = *αT*^2^/(*T* + *β*), we follow the argument that the temperature-induced shrinking of the energetic distance between valence and conduction band edge by a factor *r*(*T*) = (1 − *δ*(*T*)/*E*_G_(*T* = 0 K)) translates into the same relative shrinking of all energetic distances between states within the bandgap.

As the process of matching MPC scans recorded at different temperatures demands that both derived quantities, i.e. MPC DOS and energy scale, are temperature-independent, the mentioned temperature dependency of *E*_ωp_ − *E*_V_ is transferred to the right side of equation 5 leaving the left side with the energy scale *E*_ωp_ − *E*_V_ at *T* = 0 K:

6 $$[{E}_{\omega {\rm{p}}}-{E}_{{\rm{V}}}](T=0\,{\rm{K}})=\frac{{k}_{{\rm{B}}}T\,\mathrm{ln}([{k}_{{\rm{c}}}{N}_{{E}_{{\rm{V}}}}{k}_{{\rm{B}}}{T}^{3/2}]/\omega )}{r(T)}=\frac{{k}_{{\rm{B}}}T\,\mathrm{ln}([{k}_{{\rm{c}}}{N}_{{E}_{{\rm{V}}}}{k}_{{\rm{B}}}{T}^{3/2}]/\omega )}{1-\delta (T)/{E}_{{\rm{G}}}(T=0\,{\rm{K}})}$$

For the parameters *α* and *β* in *δ*(*T*) we used the values determined experimentally in our earlier publication^47^. While results in Fig. 2 are presented for the energy scaling in equation 6, gaps in existing research leave leeway for alternative scenarios of energy scaling. However, complementary MPC evaluations in Supplementary Section S3 relying on alternative energy scalings indeed yield the same key result of a non-constant capture coefficient for the spectrum of localised states in the bandgap.

### Composing the MPC DOS

Starting from an initial value for the product ${k}_{{\rm{c}}}{N}_{{E}_{{\rm{V}}}}$ chosen uniformly for all energy levels, this initial value might be locally too high or too low to achieve overlap of neighbouring MPC scans. The essential task of the following MPC analysis is to find the actual product $({k}_{{\rm{c}}}{N}_{{E}_{{\rm{V}}}})=\gamma \cdot {k}_{{\rm{c}}}{N}_{{E}_{{\rm{V}}}}$ to locally optimise overlap by determining the factor *γ* separately for thin slices of the MPC DOS. Considering one of these MPC DOS bins, an algorithm programmed in *MATLAB* checks for data points that describe the same MPC DOS (at least within the sufficiently narrow bin width). The found data points are most likely not scaled precisely to the same energy level by the initial ${k}_{{\rm{c}}}{N}_{{E}_{{\rm{V}}}}$ value, hence the factor *γ* has to be chosen in a way that the MPC data points found in this bin are scaled to the same energy level, Thereby, changing the product ${k}_{{\rm{c}}}{N}_{{E}_{{\rm{V}}}}$ does not only move the data points along the x-axis ([*E*_ωp_ − *E*_V_](*T* = 0 K)), but also affects the y-axis ($N({E}_{{\rm{\omega }}{\rm{p}}})/{N}_{{E}_{{\rm{V}}}}\cdot {k}_{\mu }$). The scaling algorithm includes parametrisation of MPC data points, so that the number of involved measurement points for determining the factor *γ* per bin lies in the general order of 10^2^. Further details on this MPC DOS composition procedure can be found in the Supplementary Section S4.

## Supplementary information

Supplement to: Localised states and their capture characteristics in amorphous phase-change materials

## Data Availability

The data that support the findings of this study are available from the corresponding author upon reasonable request.
