# Supplementary material for: Localised states and their capture characteristics in amorphous phase-change materials
Source: Sci Rep. 2019 Apr 29;9:6592. doi: 10.1038/s41598-019-43035-7 (PMC6488676; doi:10.1038/s41598-019-43035-7)
Supplement: Supplementary file 1 — Supplement to: Localised states and their capture characteristics in amorphous phase-change materials [file 41598_2019_43035_MOESM1_ESM.pdf]

Supplement to:
Localised states and their capture characteristics in amorphous phase-change materials

Martin Ruetten[1], Andreas Geilen[1], Abu Sebastian[2], Daniel Krebs[2], and Martin Salinga[1,*]

[1]I. Physikalisches Institut (IA), RWTH Aachen University, Sommerfeldstrasse 14, 52074 Aachen, Germany
[2]IBM Research - Zurich, Säumerstrasse 4, 8803 Rüschlikon, Switzerland
[*]Corresponding author: martin.salinga@rwth-aachen.de

Contents

S1 Details on the experimental method 2
S2 Flux-dependent MPC spectroscopy 3
S3 Conceivable energy scaling scenarios in MPC spectroscopy 5
S4 Composing the MPC DOS 5
S5 MPC spectroscopy with regard to hopping 9
S6 Extending the current MPC analysis towards multiphonon transition probabilities 12
References 17

S1 Details on the experimental method

Lateral devices were fabricated for MPC spectroscopy measurements, with 100 nm thick PCM material ($Ag_4In_3Sb_{67}Te_{26}$ or $Ge_2Sb_2Te_5$) deposited on a substrate in between two tungsten electrodes, which are 1.2 mm long and 40 μm apart. The PCM material was deposited by direct current sputtering with an LS 320 von Ardenne system at a background pressure of $2 \cdot 10^{-6}$ mbar with 20 sccm Argon flow operating in constant power mode (20 W) using stoichiometric targets of 99.99% purity. To prevent the PCM material from oxidising, it was capped *in-situ* with a 10 nm layer of $(ZnS)_{80}$:$(SiO2)_{20}$.

Electrical measurements were conducted in a Janis ST-500-2UHT cryogenic probing station, evacuated to pressures $p \leq 1 \cdot 10^{-4}$ mbar. While the device is contacted by two DC probe tips (bandwidth up to 50 MHz), a Keithley 2400 source meter is used to apply a low-field bias voltage. The device current is amplified and converted to a voltage signal by means of a Femto DHPCA-100 transimpendance amplifier, which features a switchable gain ranging from 10^2 V/A to 10^7 V/A. Using only the highest gain setting was not feasible, since with increasing gain the maximum input current of the amplifier decreases. The third essential electronic component of the setup is a HP 3562A signal analyser, which also serves as signal generator. While existing MPC spectroscopy studies commonly use a Lock-in amplifier to compare excitation signal and MPC signal (see e.g.[1]), the HP 3562A performs a single-point FFT analysis of the input signal, offering a remarkable measurement speed when measuring the amplitude of the modulated photocurrent $|I_{ac}|$ and its phase shift ϕ with respect to the excitation signal. This allows for conducting the flux-dependent MPC spectroscopy described below in a time-efficient manner. The sinusoidal source signal from the signal generator output is fed into a ThorLabs ITC4001 laserdiode controller. Apart from generating a laserdiode current proportional to the excitation signal, the ITC4001 is also used to add an offset laser current (needed to overcome the diode's lasing threshold) and to control the temperature of the Thorlabs LPS-830-FC pg-tailed laserdiode, which emits monochromatic light with $\lambda = 830$ nm (corresponding to ≈ 1.49 eV). Subsequently, the light is emitted to the optical fibre (ThorLabs HB800P), passes through an optical attenuator (type DD-600 by OZ-optics, attenuating the light in a variable range from 0 dB to 60 dB) and is fed into the cryostat chamber by means of a vacuum feed-through (OZ-optics VAC-3S3S-780-5/125-S). Great care was taken to calibrate the setup, because the frequency characteristics of laserdiode controller and the transimpedance amplifier affect the modulated photocurrent. Due to an in-house characterisation of these components, the limiting error in phase shift could be reduced below one degree.

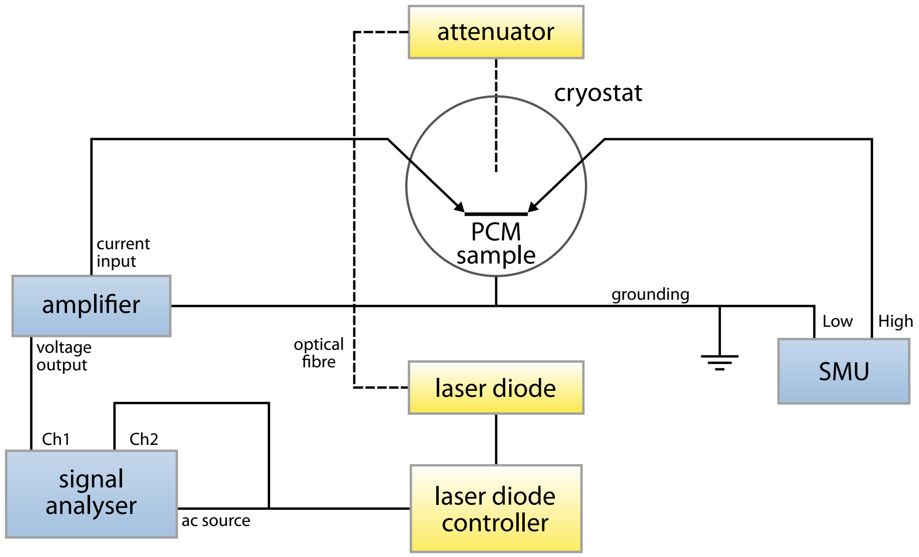

Figure 1. MPC spectroscopy setup. The scheme shows essential components of the MPC spectroscopy setup, coloured in yellow when related to the illumation of the sample and coloured in blue when related to generating and measuring electrical signals. Solid lines show electrical connections, while dashed lines represent the optical fibre.

S2 Flux-dependent MPC spectroscopy

At first sight, the question of which light flux should be used for conducting MPC scans seems redundant, because one could simply use the largest available light flux of the laserdiode to maximise the MPC signal-to-noise ratio. However, as described by work of Taylor and Simmons (e.g.[2]) and also Shockley and Read[3] on occupation statistics in the non-equilibrium steady state, increasing light flux widens the so-called recombination zone around the bandgap center, which is marked by the quasi Fermi levels for trapped electrons and holes. While a great majority of recombination traffic passes through the recombination zone, photocarriers trapped in localised states at energy levels between or near the quasi Fermi levels rather recombine instead of being re-emitted to the band and contributing to the MPC signal. Thus, a trade-off in light flux for MPC measurements has to be found, because large flux is advantageous for the signal-to-noise ratio, but can also lead to the probed energy levels being affected by recombination.

As described in the main text, a method to use flux-dependent MPC scans for probing the actual DOS is proposed. After a constant sample temperature has been set, the signal analyser performs a frequency sweep from 10 Hz to 40 kHz measuring $|I_{ac}(\omega)|$ and $\phi(\omega)$ at the highest possible light flux. Subsequently, the MPC scan is repeated with the optical attenuation level increased by 5 dB, while all other experimental parameters remain unchanged. This procedure is continued until the flux is too low to measure $|I_{ac}(\omega)|$ and $\phi(\omega)$ with a satisfactory signal-to-noise ratio. Eventually, a plot of phase shift vs. modulation frequency for various light fluxes is obtained, as displayed in the left part of figure 2. It can be observed that starting at large fluxes, the phase shift increases with decreasing flux for all modulation frequencies, meaning that all probed energy levels are affected by the recombination zone and the recorded MPC signal cannot be used for DOS spectroscopy. However, further lowering the flux causes saturation in phase shift, starting at high frequencies. These high frequencies correspond to the energy levels closer to the mobility edge, which are consequentially the first probed levels leaving the recombination zone upon flux decrease. Feeding the saturated, flux-independent phase shift to the expressions for DOS spectroscopy described in the main text yields an MPC DOS which is related to the actual DOS. Further decreasing the flux results in more frequencies that can be used for MPC spectroscopy, but it also impairs the signal-to-noise ratio. Looking at the exemplary phase shift data from figure 2, it gets clear that a compromise between noise and range of usable frequencies has to be found. As described above, the same fluxes and frequencies, that imply a saturation in phase shift, lead to the ratio $|I_{ac}|/F_{ac}$ being flux-independent, as it can be seen in the right panel of figure 2. To conclude, conducting MPC scans with varying flux eventually identifies the suitable flux and frequency range that allows for probing the actual DOS, unaffected by recombination. However, this method can only be applied as long as the MPC accessible range is limited by the flux-dependent quasi Fermi level. As soon as the MPC accessible range is limited by the dark Fermi level at elevated temperatures, the approach presented in this subsection is not applicable any more.

To perform a complete MPC experiment, sample temperatures in steps of 5 K are set sequentially and the procedure described above is repeated for each temperature. Eligible fluxes at a specific temperature can be estimated based on the data from the previous temperature step, so that it is often sufficient to record two or three MPC scans instead of seven as shown in figure 2 at varying flux. However, it should be stressed that the overall series of MPC spectroscopy measurements is still time-consuming and that only using the HP3652A signal analyser makes this flux-dependent MPC spectroscopy practicable. To handle the considerable amount of data per temperature step and to quickly determine the usable frequency range, a graphical user interface programmed in *MATLAB* including the necessary calibration information for phase and amplitude was found helpful.

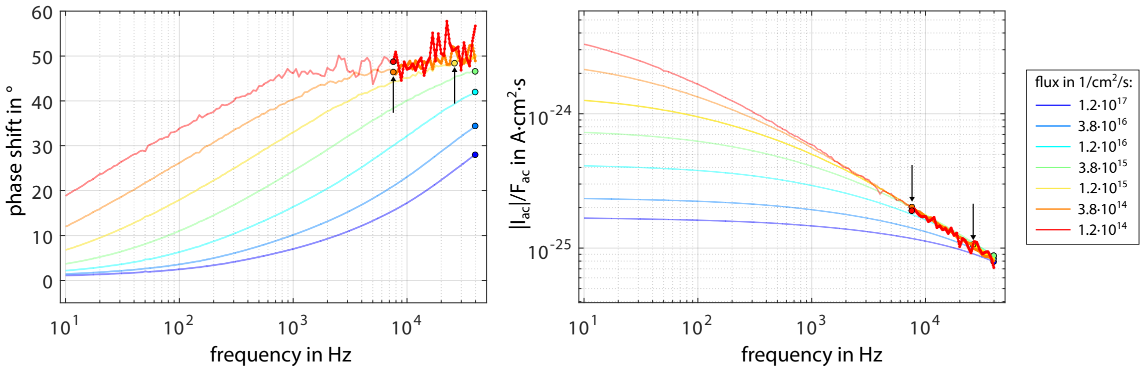

Figure 2. MPC scans at constant temperature upon flux variation. Exemplary MPC scans on amorphous $Ge_2Sb_2Te_5$ at $T = 160\,K$ are shown to illustrate the procedure of finding data unaffected by the recombination zone. **Left:** Frequency sweeps recorded with gradually increased optical attenuation reveal that the phase shift is flux-independent for lower fluxes, starting at high frequencies corresponding to probed energy levels closer to the mobility edge. These energy levels are not affected by the recombination zone and the corresponding phase shift data (plotted bold and the transition frequency marked by arrows) is used for DOS spectroscopy. Note that lagging phase shift is defined as positive in accordance with existing MPC literature[4]. **Right:** The same frequencies and fluxes, that yield a flux-independent phase shift, imply also a flux-independent ratio $|I_{ac}|/F_{ac}$. Consequently, the MPC signal and the resulting DOS probed by MPC spectroscopy does not change upon further decreasing flux.

S3 Conceivable energy scaling scenarios in MPC spectroscopy

While the Varshni model[5] is commonly used in PCM research to describe the shrinkage of the bandgap $E_G(T) = E_C(T) - E_V(T)$ upon heating, gaps in existing studies leave leeway for multiple scenarios on how localised states in the amorphous phase could be affected by temperature variation. Following the approaches of e.g. Kaes et al.[6] and Luckas et al.[7], one possible scenario involves that localised states keep their relative position within the bandgap. Since their movement is proportional to the movement of the band edges upon heating, this scenario is often called *pro rata* and equals the approach applied in the main text. Alternatively, theoretical studies on crystalline semiconductors suggest that the entire bands move towards each other[8,9]. Transfering this *bandshifting* concept to the amorphous phase yields a stronger temperature dependence of the localised states compared to the pro rata scenario. As a third conceivable approach, Spear et al.[10,11] complement the Anderson localisation model by the effect of temperature and understand the bandgap characteristics as sole movement of the mobility edges (*mobility edge shifting*), leaving localised states in between unaffected upon temperature variation.

Starting out from these three alternative concepts on the temperature dependence of localised states in the bandgap, the corresponding energy scaling expressions for MPC spectroscopy shall be developed briefly. With respect to the main text, it is convenient to start from the expression without any temperature dependence of localised states:

$$E_{\omega\text{p}} - E_\text{V} = k_\text{B} T \ln \left(\left[k_\text{c} N_{E_\text{V}} k_\text{B} T^{3/2} \right] / \omega \right) . \tag{1}$$

To begin with the simplest case, the bandshifting approach is already described by equation 1 and the temperature dependence of the mobility edge is simply not included in this scenario, since it does not affect the MPC-relevant energetic distance to the probed energy levels. In contrast to this, the mobility edge shifting approach transfers the temperature dependence of the bandgap to $\left[E_{\omega\text{p}} - E_\text{V}\right](T)$. So far, no published results can be found on whether the shrinkage of the bandgap with increasing temperature originates from both bandedges moving in an equal measure or from one bandedge dominating the effect. In view of this lack of knowledge, one can e.g. assume that the shrinkage of the bandgap is equally distributed to both bandedges, implying that each bandegde is affected by half of the Varshni contribution. By means of the Varshni model $E_G(T) = E_G(T = 0\,\text{K}) - \delta(T)$ with $\delta(T) = \alpha T^2/(T+\beta)$[5], equation 1 reads as

$$\left[E_{\omega\text{p}} - E_\text{V}\right](T = 0\,\text{K}) = k_\text{B} T \ln \left(\left[k_\text{c} N_{E_\text{V}} k_\text{B} T^{3/2} \right] / \omega \right) + \frac{1}{2} \cdot \frac{\alpha T^2}{T+\beta} . \tag{2}$$

As third scenario, the pro rata approach proposes that the temperature-induced shrinking of the energetic distance between valence- and conduction bandedge by a factor $r(T) = (1 - \delta(T)/E_G(T = 0\,\text{K}))$ (with $\delta(T) = \alpha T^2/(T+\beta)$) translates into the same relative shrinking of all energetic distances between states within the bandgap. Referring to the derivation given in the main text, the pro rata energy scale is eventually given by

$$\left[E_{\omega\text{p}} - E_\text{V}\right](T = 0\,\text{K}) = \frac{k_\text{B} T \ln \left(\left[k_\text{c} N_{E_\text{V}} k_\text{B} T^{3/2} \right] / \omega \right)}{r(T)} = \frac{k_\text{B} T \ln \left(\left[k_\text{c} N_{E_\text{V}} k_\text{B} T^{3/2} \right] / \omega \right)}{1 - \delta(T)/E_G(T = 0\,\text{K})} . \tag{3}$$

All equations for the different energy scaling scenarios include the product $k_\text{c} N_{E_\text{V}}$, which is unknown in the first instance and determined during composing the coherent DOS, as it is described in the main text and the supplement. Thus, $k_\text{c} N_{E_\text{V}}$ plays the same role in the here presented alternative analysis as the traditional attempt-to-escape frequency plays in the analysis of Longeaud et al.[4].

While the results presented in the main text are based on the pro rata energy scaling, our key result of non-constant capture characteristics along the continuous spectrum of localised states can be also investigated for the band shifting and mobility edge shifting approach to account for the incomplete knowledge on the actual temperature dependence of localised states. Figure 3 displays the results shown in the main text on amorphous $Ge_2Sb_2Te_5$ and $Ag_4In_3Sb_{67}Te_{26}$ together with the analysis outcome based on the band shifting and mobility edge shifting approach. As can be seen, our qualitative finding of non-constant capture coefficients is preserved upon changing between alternative energy scalings.

S4 Composing the MPC DOS

As described in the main text, the essential task of the proposed MPC analysis is to find the actual product $(k_\text{c} N_{E_\text{V}})' = \gamma \cdot k_\text{c} N_{E_\text{V}}$ to locally optimise overlap by determining the factor γ separately for thin slices of the MPC DOS. Considering one of these MPC DOS bins, an algorithm programmed in *MATLAB* checks for any data points within this bin. Approaching the scaling mathematically begins with parametrising the MPC data points recorded for the same temperature within and to some extend also around the considered MPC DOS bin by a linear function g, which is motivated by the locally straight behaviour of the MPC scans (see figure 4) and helps to reduce the effect of noise. Although the data are plotted as $\ln\left(N(E_{\omega\text{p}})/N_{E_\text{V}} \cdot k_\mu\right)$ vs. $\left[E_{\omega\text{p}} - E_\text{V}\right](T = 0\,\text{K})$,

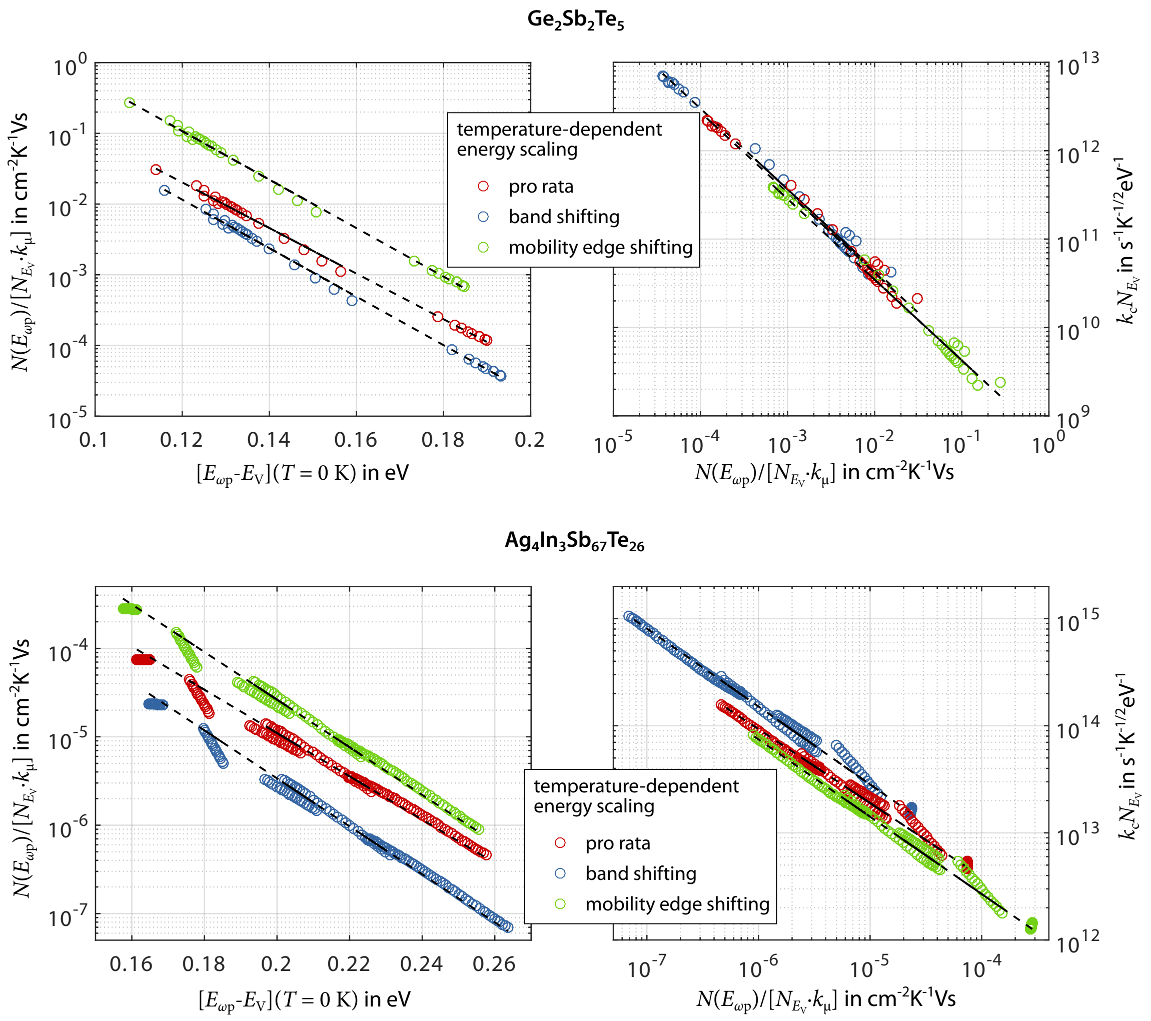

Figure 3. MPC analysis outcome on the continuous spectrum of localised states in amorphous $Ge_2Sb_2Te_5$ and $Ag_4In_3Sb_{67}Te_{26}$ for various energy scaling scenarios. To take into account the incomplete knowledge on the temperature dependence of localised states, the MPC analysis outcome presented in the main text (see figure 2) for the pro rata energy scaling is plotted together with the outcome using the band shifting and mobility edge shifting approach for energy scaling. While changing the energy scaling does effect absolute numbers regarding MPC DOS and energy, the qualitative phenomenon of non-constant capture coefficients along the continuous spectrum of localised states does not depend on the chosen energy scaling.

it turns out to be convenient to write g as a function of $\ln\left(N(E_{\omega\mathrm{p}})/N_{E_\mathrm{V}} \cdot k_\mu\right)$. Considering two neighbouring MPC scans at temperatures T_1 and T_2 as illustrated in the lower part of figure 4, one can write

$$g_1\left(\ln\left(\left.\frac{N(E_{\omega\mathrm{p}})}{N_{E_\mathrm{V}} \cdot k_\mu}\right|_{T_1}\right)\right) = p_1 \cdot \ln\left(\left.\frac{N(E_{\omega\mathrm{p}})}{N_{E_\mathrm{V}} \cdot k_\mu}\right|_{T_1}\right) + q_1 = \left\{\left[E_{\omega\mathrm{p}} - E_\mathrm{V}\right](T = 0\,\mathrm{K})\right\}_{T_1} \tag{4}$$

$$g_2\left(\ln\left(\left.\frac{N(E_{\omega\mathrm{p}})}{N_{E_\mathrm{V}} \cdot k_\mu}\right|_{T_2}\right)\right) = p_2 \cdot \ln\left(\left.\frac{N(E_{\omega\mathrm{p}})}{N_{E_\mathrm{V}} \cdot k_\mu}\right|_{T_2}\right) + q_2 = \left\{\left[E_{\omega\mathrm{p}} - E_\mathrm{V}\right](T = 0\,\mathrm{K})\right\}_{T_2} . \tag{5}$$

As next step, it is useful to calculate how multiplying the product $k_\mathrm{c}N_{E_\mathrm{V}}$ with a factor γ to obtain $\left(k_\mathrm{c}N_{E_\mathrm{V}}\right)' = \gamma \cdot k_\mathrm{c}N_{E_\mathrm{V}}$ affects the MPC DOS axis and energy axis. Regarding the former one, the equation given in the main text yields

$$\ln\left(\left.\frac{N(E_{\omega\mathrm{p}})}{N_{E_\mathrm{V}} \cdot k_\mu}\right|_{\left(k_\mathrm{c}N_{E_\mathrm{V}}\right)'}\right) = \ln\left(\left.\frac{N(E_{\omega\mathrm{p}})}{N_{E_\mathrm{V}} \cdot k_\mu}\right|_{k_\mathrm{c}N_{E_\mathrm{V}}}\right) - \ln(\gamma) , \tag{6}$$

while the pro rata energy scaling (see equation 3) is affected according to

$$\left\{\left[E_{\omega\mathrm{p}} - E_\mathrm{V}\right](T = 0\,\mathrm{K})\right\}_{\left(k_\mathrm{c}N_{E_\mathrm{V}}\right)'} = \left\{\left[E_{\omega\mathrm{p}} - E_\mathrm{V}\right](T = 0\,\mathrm{K})\right\}_{k_\mathrm{c}N_{E_\mathrm{V}}} + \frac{\ln(\gamma) \cdot k_\mathrm{B}T}{1 - \left[\alpha T^2/T + \beta\right]/E_\mathrm{G}(T = 0\,\mathrm{K})} . \tag{7}$$

It should be noted that for alternative energy scaling scenarios (bandshifting or mobility edge shifting), equation 7 varies accordingly. Furthermore, it is noteworthy that the shift along the MPC DOS axis caused by the multiplication with γ is independent of temperature, while the shift along the energy axis increases with increasing temperature. Moreover, if the starting value of $k_\mathrm{c}N_{E_\mathrm{V}}$ was initially chosen too large, requiring correction with $\gamma < 1$ (and $\ln(\gamma) < 0$), the MPC data points are scaled towards a higher MPC DOS and lower energy (as the scenario shown in figure 4 qualitatively demonstrates). It is assumed in the following that the temperature dependence of γ is weak and the approximation of $\gamma(T_1) \approx \gamma(T_2) = \gamma$ is valid, which is only a small constraint in the present study, since the data investigated have a temperature step size of $|T_1 - T_2| = 5\,\mathrm{K}$. Mathematically expressing the demand for matching the energy position of MPC data points recorded at temperatures T_1 and T_2 within one specific MPC DOS-bin leads to

$$\begin{aligned} g_1\left(\ln\left(\left.\frac{N(E_{\omega\mathrm{p}})}{N_{E_\mathrm{V}} \cdot k_\mu}\right|_{T_1}\right)\right) &+ \frac{\ln(\gamma) \cdot k_\mathrm{B}T_1}{1 - \left[\alpha T_1^2/T_1 + \beta\right]/E_\mathrm{G}(T = 0\,\mathrm{K})} = \\ g_2\left(\ln\left(\left.\frac{N(E_{\omega\mathrm{p}})}{N_{E_\mathrm{V}} \cdot k_\mu}\right|_{T_2}\right)\right) &+ \frac{\ln(\gamma) \cdot k_\mathrm{B}T_2}{1 - \left[\alpha T_2^2/T_2 + \beta\right]/E_\mathrm{G}(T = 0\,\mathrm{K})} , \end{aligned} \tag{8}$$

and solving for $\ln(\gamma)$ eventually yields

$$\ln(\gamma) = \frac{\left\{\left[E_{\omega\mathrm{p}} - E_\mathrm{V}\right](T = 0\,\mathrm{K})\right\}_{T_2} - \left\{\left[E_{\omega\mathrm{p}} - E_\mathrm{V}\right](T = 0\,\mathrm{K})\right\}_{T_1}}{k_\mathrm{B} \cdot E_\mathrm{G}(T = 0\,\mathrm{K}) \cdot \left(\frac{T_1}{E_\mathrm{G}(T = 0\,\mathrm{K}) - \left[\alpha T_1^2/T_1 + \beta\right]} - \frac{T_2}{E_\mathrm{G}(T = 0\,\mathrm{K}) - \left[\alpha T_2^2/T_2 + \beta\right]}\right)} . \tag{9}$$

For the complete analysis, bin after bin is investigated, and after parametrising suitable MPC data points, equation 9 determines the $\ln(\gamma)$ values for rescaling the data points which are supposed to match. In case MPC data points belonging to more than two different temperatures (e.g. T_1, T_2 and T_3) are found in one bin, the above described procedure is first conducted for data points from T_1 and T_2 and subsequently for data points from T_2 and T_3, which results in a temperature-dependent γ for this particular bin. However, this case occurs only very rarely within the here presented data sets. Apart from this aspect, it was pointed out above that the MPC DOS bins need to be sufficiently thin, while it is a priori not clear what sufficiently thin means. Thus, the analysis of the data set is once conducted with dividing the MPC DOS data in 100 logarithmically spaced bins. Subsequently, this amount of bins is increased by 100, the analysis is repeated and the outcome is compared to the outcome of the first analysis. This procedure is continued until the analysis outcome does not change any more upon increasing bin number, yielding the sufficiently thin bin width.

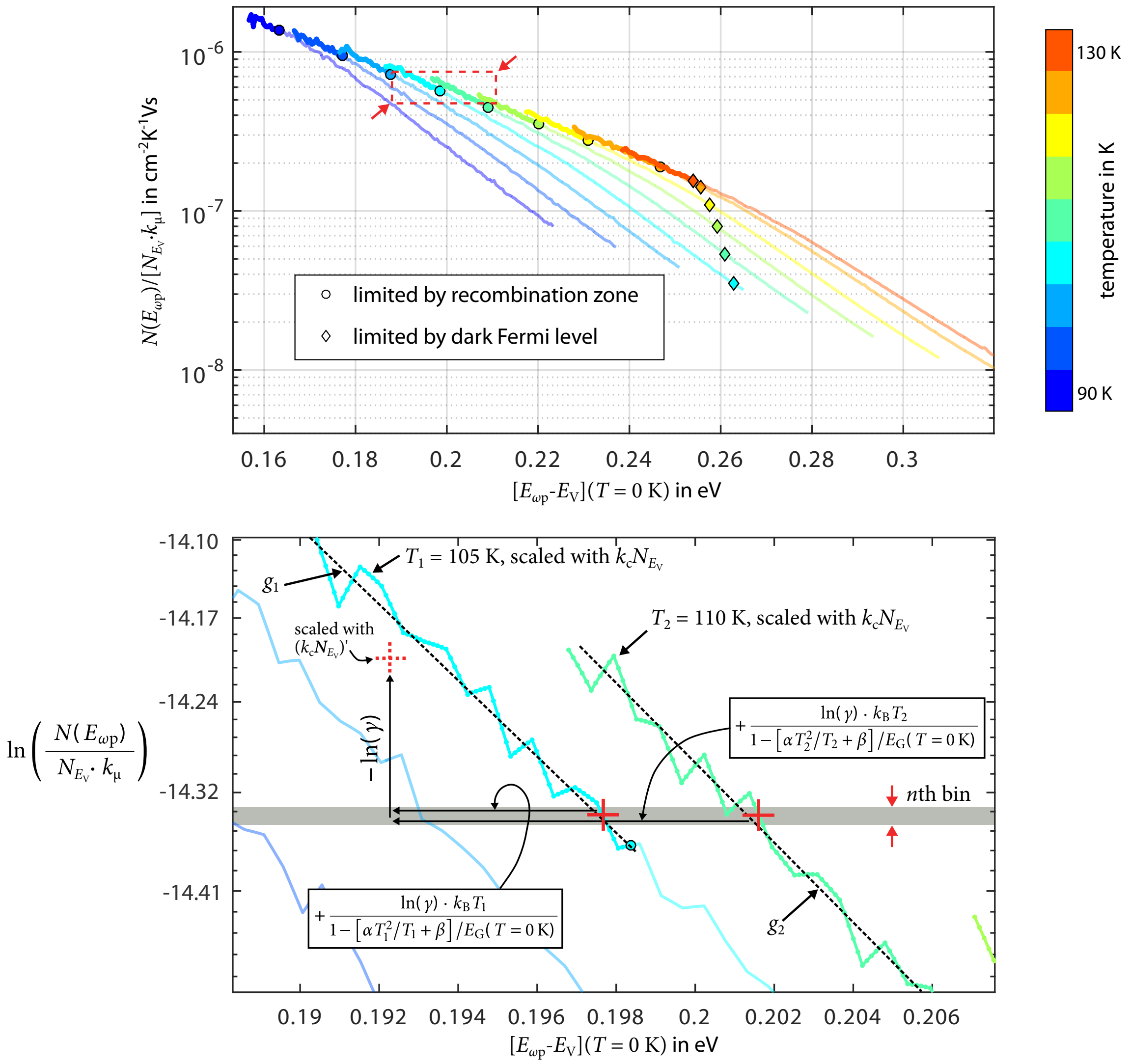

Figure 4. MPC analysis of exemplary data on amorphous $Ag_4In_3Sb_{67}Te_{26}$. Top: MPC scans recorded at temperatures between 90 K and 130 K (in 5 K steps) are plotted with a starting value for $k_c N_{E_V} = 1 \cdot 10^{15}\,\mathrm{s^{-1}K^{-1/2}eV^{-1}}$. The x-axis is given by the energy scaling, and the y-axis (according to the expression given in the main text) is proportional to the DOS (also called *MPC DOS*). Data points that are identified as unaffected by the recombination zone are plotted bold, and the limits are marked by circles. Additionally, an estimate for the dark Fermi level based on results from[12] as ultimate limit of the MPC accessible energy levels is marked by diamonds. However, it should be stressed that due to the arbitrary starting value of $k_c N_{E_V}$, absolute numbers of energy and also MPC DOS are not significant at this first analysis step. The goal of the MPC analysis is to compose a coherent picture of the MPC DOS out of the thick-plotted MPC data, by matching the MPC DOS of energy levels that are probed at different temperatures. To achieve these overlaps of MPC scans, the appropriate product $(k_c N_{E_V})' = \gamma \cdot k_c N_{E_V}$ needs to be determined. The according procedure is shown exemplary for data in the red-framed area in the bottom part of the figure. **Bottom:** The MPC DOS data is divided into logarithmically spaced bins, and the algorithm checks each bin for MPC data of two different temperatures T_1 and T_2, which are supposed to be scaled to the same energy level. If it makes a find (marked by solid red crosses), the corresponding data is firstly parametrised (g_1,g_2) to reduce the effect of noise and secondly scaled to match in MPC DOS and energy scaling. In the shown example, the starting value of $k_c N_{E_V}$ turns out to be too high, leading to $\gamma < 0$, so that both MPC data points are shifted to lower energy and higher MPC DOS. The bin width and the vertical and horizontal arrows indicating the shift are only drawn qualitatively for illustration. A real bin is thinner and the two data points (red crosses) are actually scaled to the coordinates (0.134 eV, $4.9 \cdot 10^{-4}\,\mathrm{cm^{-2}K^{-1}Vs}$).

S5 MPC spectroscopy with regard to hopping

As mentioned in the main text, the basic MPC equations known from Longeaud[13] have been developed for band transport as dominating electrical transport mechanism in terms of photocarriers, which however does not imply any restriction with respect to the modulated photoconductivity data recorded for the present work. This statement shall be explained in the following, because especially with respect to the MPC scans recorded at low temperatures, one might wonder whether it is justified to neglect the effect of hopping - meaning that charge carriers in localised states move from one localised state to the next without being re-emitted to the band - as alternative electrical transport mechanism. Previous studies (for example[14]) suggest that hopping dominates darkconductivity (σ_{dark}) in amorphous PCM only for temperatures below about 160 K. In the case of darkconductivity, a pronounced kink in the σ_{dark} vs. $1/T$ plot, caused by a change in activation energy for electrical conduction, is typically related to the onset of hopping. However, it has to be noted that detecting signs of hopping in darkconductivity data does by no means imply that photoconductivity is affected by hopping as well. Referring to figure 5, darkconductivity data recorded during the MPC spectroscopy measurement series for the present work display a slight kink at low temperatures for both materials, which could be interpreted as onset of hopping transport. However, corresponding dc photoconductivity data lack such a kink at low temperatures. Based on this observation, we cannot report any indication of a change in the electrical transport mechanism towards low temperatures with respect to photocarriers.

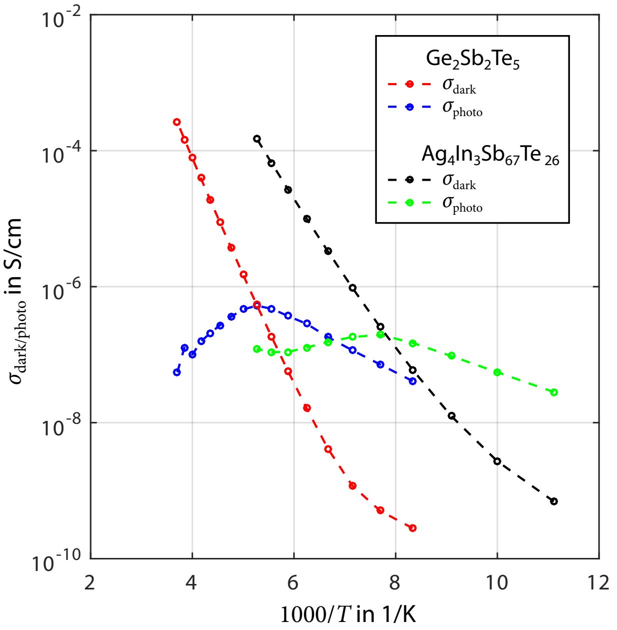

Figure 5. Temperature-dependent dark- and photoconductivity data recorded on amorphous $Ag_4In_3Sb_{67}Te_{26}$ and $Ge_2Sb_2Te_5$. The shown dark- and photoconductivity data have been recorded in the course of the MPC spectroscopy measurement series for the present work and thus have been performed on the same samples as the MPC measurements presented in the main text (photoconductivity data are shown for a dc flux of $1.2 \cdot 10^{16}\,\mathrm{cm^{-2}s^{-1}}$). While darkconductivity data display a slight kink at low temperatures for both materials, which could be interpreted as onset of hopping transport, corresponding dc photoconductivity data lack such a kink at low temperatures.

The absence of a kink in low-temperature photoconductivity data as described above might already be seen as evidence that MPC measurements in the present work are not affected by a change in transport mechanism. For the sake of completeness, we point out that it is not at all straightforward to determine how a potential change in electrical transport mechanism from band transport to hopping would affect modulated photoconductivity measurements. In literature, a comprehensive study by Longeaud and Tobbeche[13] can be found, which investigates the influence of hopping on photoconductivity. The essential conclusions from this work with respect to MPC spectroscopy are outlined below, further explaining why the MPC measurements conducted in this work do not provide evidence of dominating hopping transport.

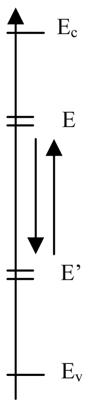

Figure 6. Scheme of transition between two energy levels. To investigate the influence of hopping on photoconductivity, Longeaud and Tobbeche[13] propose the modification of the occupation function by considering states of the same species at two energy levels E and E'. The occupation of these levels can not only change by capturing and emission with respect to the band as described in the original work on non-equilibrium steady-state statistics[2,3], but also due to hopping between the two energy levels. Reprinted from[13].

Starting from the theoretical framework developed by Shockley and Read[3] and Taylor and Simmons (for example[2]) to describe the occupation of localised states in the non-equilibrium steady state, it is obvious that taking hopping into account requires a modification of the occupation function $f(E)$. The traditional version of $f(E)$ only considers the processes of capturing and emission with regard to the band to change the occupation of a state. Longeaud and Tobbeche approach this modification of $f(E)$ by describing hopping between states at two energy levels E and E' (see also figure 6), which belong per definition to the same species. A change in occupation of a state at level E due to hopping is thus given by a carrier hopping down to a state at level E', or a carrier from a state at level E' hopping up to a state at level E. It therefore reads

$$\frac{\partial f_{\mathrm{hop}}(E)}{\partial t} = \underbrace{(1-f(E))\int_{E_\mathrm{V}}^{E_\mathrm{C}} \Gamma_{E',E} N(E') f(E') \mathrm{d}E'}_{\text{hopping up: E}'\to\text{E}} - \underbrace{f(E)\int_{E_\mathrm{V}}^{E_\mathrm{C}} \Gamma_{E,E'} N(E')\left(1-f(E')\right)\mathrm{d}E'}_{\text{hopping down: E}\to\text{E}'} \tag{10}$$

including the hopping probability Γ, which depends on the mean intersite spacing r_E, the decay length of the wavefunction and the so-called attempt-to-hop frequency ν_0 as proposed by Marshall[15]. The overall change in occupation is thus given by

$$\frac{\partial f(E)}{\partial t} = (1-f(E))\int_{E_\mathrm{V}}^{E_\mathrm{C}} \Gamma_{E',E} N(E') f(E') \mathrm{d}E' - f(E)\int_{E_\mathrm{V}}^{E_\mathrm{C}} \Gamma_{E,E'} N(E')\left(1-f(E')\right)\mathrm{d}E'$$
$$+\underbrace{\bar{n}+e_\mathrm{p}(E)-f(E)\left[\bar{n}+\bar{p}+e_\mathrm{n}(E)+e_\mathrm{p}(E)\right]}_{\text{Shockley-Read/Taylor-Simmons statistics}}, \tag{11}$$

where the second part is given by the original occupation statistics excluding hopping (all quantities are described in[13]).

After modifying the non-equilibrium steady state occupation function according to equation 11, the following procedure is identical to what is described in the original work by Longeaud[4] for the MPC analysis without considering any hopping. This means that the rate equations are used to calculate the concentration of free electrons n and holes p, by solving $\partial f/\partial t = \partial p/\partial t = \partial n/\partial t = 0$. While the numerical calculation of n and p by means of equation 11 is still feasible, an analytical expression to relate the amplitude of the modulated photocurrent $|I_\mathrm{ac}|$ and phase shift ϕ to the DOS as given by equation the main text cannot be obtained any more[13]. Therefore, instead of developing a new mathematical description to analyse MPC spectroscopy results dominated by hopping as transport mechanism, the analysis by Longeaud and Tobbeche rather focusses on investigating under which conditions the traditional, band transport based MPC analysis breaks down. To do so, they use various DOS typical for hydrogenated amorphous silicon as input for numerically calculating the modulated components of n and p, providing simulated results for $|I_\mathrm{ac}|$ and ϕ once with and once without considering hopping. It should be noted that the step of obtaining the modulated photocurrent including hopping is slightly more complicated compared the scenario without hopping, because the photoconductivity given by the transport of carriers through extended states has to be complemented by hopping transport:

$$\sigma = \underbrace{qn\mu_{\mathrm{ext,n}}n + q\mu_{\mathrm{ext,p}}}_{\text{via extended states}}$$
$$+ \frac{q^2}{6k_\mathrm{B}T}\int_{E_\mathrm{V}}^{E_\mathrm{C}} N(E)f(E)\int_{E_\mathrm{V}}^{E_\mathrm{C}} \Gamma_{E,E'} N(E')\cdot\left(1-f(E')\right) r_\mathrm{E}^2 \mathrm{d}E'\mathrm{d}E\,. \tag{12}$$

Conductivity via hopping transport in equation 12 is given by hopping diffusivity and the Einstein equation, which is only valid at low electrical fields and when the field does not alter the probabilites of upward hopping (see for example[16]).

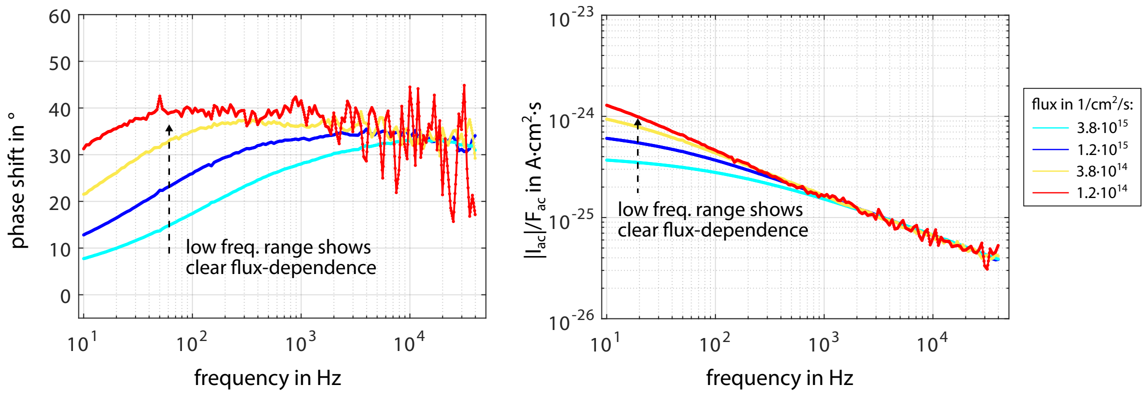

Figure 7. Inspecting MPC scans with respect to hopping transport. To look for evidence whether hopping dominates the MPC signal, the low temperature MPC scans recorded at 90 K on amorphous $Ag_4In_3Sb_{67}Te_{26}$ are examined with respect to flux dependence in ϕ and $|I_{ac}|/F_{ac}$. At high frequencies, both quantities are flux-independent, because the probed energy levels are unaffected by the recombination zone. If hopping is the dominant transport mechanism, Longeaud and Tobbeche suggest that MPC scans are flux independent at low frequencies, which is clearly not observed.

After $|I_{ac}|$ and ϕ have been numerically calculated - with and without considering hopping - the outcome is fed into the traditional MPC DOS expression. By comparing the resulting MPC DOS with the original DOS input it is checked, under which conditions the traditional MPC DOS expression fails to reproduce the DOS. As fingerprint of hopping in MPC scans, Longeaud and Tobbeche observe an underestimation of the DOS at low temperatures, starting at low frequencies. They also give an illustrative explanation for this underestimation, originating from an overestimated MPC amplitude $|I_{ac}|$. As explained by Longeaud[4], the MPC signal is controlled by carriers being re-emitted from states whose emission rate equals the modulation frequency. Carriers trapped in states located at energies deeper in the band exhibit a much lower emission rate and are thus considered as *frozen in*[13]. With respect to band transport, these frozen in carriers cannot contribute to the MPC signal, but they certainly can by means of hopping transport. For lower temperatures during MPC recording, probed energy levels are located closer to the mobility edge and more carriers are frozen in, potentially increasing $|I_{ac}|$ by hopping. Consequently, the underestimation of the DOS due to hopping transport starts at low temperatures. The fact that the underestimation appears first at low frequencies could be explained by the increased time that trapped carriers have to find a suitable hopping site and to contribute to the MPC signal.

In addition to their simulation outcome and corresponding, illustrative explanations, Longeaud and Tobbeche also suggest an experimental procedure to detect the effect of hopping and exclude affected data from further DOS analysis. They observe that the above described underestimation of the DOS resulting from hopping is very similar to the DOS underestimation resulting from probing states at energy levels in the recombination zone. While both phenomena look the same when occurring in an MPC DOS vs. energy plot, the latter one can be remediated by decreasing the light flux, as long as the signal-to-noise ratio is sufficient. On the contrary, the DOS underestimation due to hopping remains unaffected by variation of the generation rate. Longeaud and Tobbeche therefore suggest in the event of an obvious MPC DOS underestimation to vary the flux and to check the MPC DOS data (ϕ and $|I_{ac}|/F_{ac}$) for flux dependence. Strictly speaking, this approach is not straightforward, because flux independence of ϕ and $|I_{ac}|/F_{ac}$ can consequentially have two reasons, namely a) hopping is dominating the transport mechanism for photoconductivity or b) the recorded MPC signal is unaffected by recombination. Only a) results in an DOS underestimation, which can only be identified when the coherent MPC DOS is composed of various MPC scans recorded at different temperatures.

Investigating the MPC data from this work with respect to the approach of Longeaud and Tobbeche, clear evidence against hopping as dominant transport mechanism can be found. This is exemplarily explained by means of flux-dependent low temperature MPC scans recorded at 90 K on amorphous $Ag_4In_3Sb_{67}Te_{26}$ (see figure 7). In these MPC scans, flux independence actually occurs, but it is observed only for high frequencies and is therefore ascribed to the probed energy levels being unaffected by recombination. At low frequencies - which is the regime where the effect of hopping should be strongest according to Longeaud and Tobbeche - ϕ and $|I_{ac}|/F_{ac}$ clearly depend on flux. The same observation is made for low-temperature MPC data on amorphous $Ge_2Te_2Te_5$, serving the overall conclusion that band transport based MPC analysis is sufficient to analyse the MPC data presented in this work.

S6 Extending the current MPC analysis towards multiphonon transition probabilities

As described in the main text, it appears as a promising next step to extend the MPC analysis developed by Longeaud[4] towards the multiphonon interpretation of charge carrier transitions. This implies an exponential energy dependence of the capture coefficient, while the original MPC framework relies on the assumption of $c_{\mathrm{p/n}} = \text{const.}$ among all involved localised states. The following should be seen as sketch of how the existing evaluation of experimental MPC data could be modified in order to implement said exponential energy dependence of $c_{\mathrm{p/n}}$.

Following Baranovskii's approach[17–19], the probability $P_{\downarrow}$ for a carrier transition from the band to a localised state having a multiphonon nature is given by

$$P_{\downarrow}(E) = \nu \cdot \exp\left(\frac{-\gamma E}{\hbar\omega_0}\right), \tag{13}$$

wherein ν is treated as constant prefactor and E denotes the energetic distance between the band and the localised state. The parameter γ is close to unity and exhibits a weak logarithmic dependence on energy, which is neglected in the following. Furthermore, it is assumed that E is considerably greater than the characteristic phonon energy $\hbar\omega_0$. On the basis of equation 13, the escape probability from the localised state back to the band can be written as $P_{\uparrow}(E) = P_{\downarrow}(E) \cdot \exp(-E/k_{\mathrm{B}}T)$[17]. Incorporating $P_{\uparrow}(E)$ and $P_{\downarrow}(E)$ in the original capture coefficient $c_{\mathrm{n/p}}$ for electrons/holes as they can be found for example in the work of Longeaud[4] yields:

$$\begin{aligned} c_{\mathrm{n}}(E) &= \sigma_{\mathrm{n}} \cdot \upsilon_{\mathrm{th}} \cdot \exp\left(-[E_{\mathrm{C}} - E]/\hbar\omega_0\right) \\ c_{\mathrm{p}}(E) &= \sigma_{\mathrm{p}} \cdot \upsilon_{\mathrm{th}} \cdot \exp\left(-[E - E_{\mathrm{V}}]/\hbar\omega_0\right), \end{aligned} \tag{14}$$

with $\sigma_{\mathrm{n/p}}$ denoting the capture cross section for electrons/holes and υ_{th} denoting the thermal velocity. Within the notation used by Longeaud, the constant capture rates $\bar{n} = n \cdot c_{\mathrm{n}}$ and $\bar{p} = p \cdot c_{\mathrm{p}}$ turn into energy-dependent capture rates $\bar{n}(E) = n \cdot c_{\mathrm{n}}(E)$ and $\bar{p}(E) = p \cdot c_{\mathrm{p}}(E)$. In a similar manner, the emission rates of electrons/holes $e_{\mathrm{n/p}}$ have to be revised:

$$\begin{aligned} e_{\mathrm{n}}(E) &= N_{\mathrm{C,eff}} \cdot \sigma_{\mathrm{n}} \cdot \upsilon_{\mathrm{th}} \cdot \exp\left(-[E_{\mathrm{C}} - E]/\hbar\omega_0\right) \cdot \exp\left(-[E_{\mathrm{C}} - E]/k_{\mathrm{B}}T\right) \\ e_{\mathrm{p}}(E) &= N_{\mathrm{V,eff}} \cdot \sigma_{\mathrm{p}} \cdot \upsilon_{\mathrm{th}} \cdot \exp\left(-[E - E_{\mathrm{V}}]/\hbar\omega_0\right) \cdot \exp\left(-[E - E_{\mathrm{V}}]/k_{\mathrm{B}}T\right), \end{aligned} \tag{15}$$

including the effective density of states at the valence/conduction bandedge $N_{\mathrm{V/C,eff}}$. Taking account of these modified expressions for the capture coefficients as well as the trapping rates and emission rates, the derivation steps in the paper of Longeaud[4] are inspected in the following paragraphs. Some of these steps might still be valid under the additional energy dependencies, while others might need to be adapted.

To begin with, three rate equations form the basis for deriving values of the free charge carrier concentrations n and p. Considering the expressions introduced above, these rate equations read as follows:

$$\begin{aligned} \frac{\mathrm{d}n}{\mathrm{d}t} &= G - \int_{E_{\mathrm{V}}}^{E_{\mathrm{C}}} \bar{n}(E)N(E)\left[1 - f(E)\right]\mathrm{d}E + \int_{E_{\mathrm{V}}}^{E_{\mathrm{C}}} e_{\mathrm{n}}(E)N(E)f(E)\mathrm{d}E \\ \frac{\mathrm{d}p}{\mathrm{d}t} &= G - \int_{E_{\mathrm{V}}}^{E_{\mathrm{C}}} \bar{p}(E)N(E)f(E)\mathrm{d}E + \int_{E_{\mathrm{V}}}^{E_{\mathrm{C}}} e_{\mathrm{p}}(E)N(E)\left[1 - f(E)\right]\mathrm{d}E \\ \frac{\mathrm{d}f}{\mathrm{d}t} &= \bar{n}(E) + e_{\mathrm{p}}(E) - f(E)\left[\bar{n}(E) + \bar{p}(E) + e_{\mathrm{n}}(E) + e_{\mathrm{p}}(E)\right], \end{aligned} \tag{16}$$

with t denoting the time, $N(E)$ the DOS at energy E, $f(E)$ the occupation function and G the generation rate of free carriers. In line with the original derivation, solving these rate equations for n and p can be approached by separating the dc contribution from the ac contribution, which yields $n = n_{\mathrm{dc}} + n_{\mathrm{ac}}\exp(i\omega t)$ and $p = p_{\mathrm{dc}} + p_{\mathrm{ac}}\exp(i\omega t)$, wherein i is the imaginary number. Similarly, the generation rate and the occupation function can be split up into their respective dc and ac parts. Further writing $n_{\mathrm{ac}} = n_{\mathrm{r}} + i n_{\mathrm{i}}$ and $p_{\mathrm{ac}} = p_{\mathrm{r}} + i p_{\mathrm{i}}$ leads to a linear system of four equations:

$$A_{\mathrm{n}}n_{\mathrm{i}} + A_{\mathrm{p}}p_{\mathrm{i}} + B_{\mathrm{n}}n_{\mathrm{r}} - B_{\mathrm{p}}p_{\mathrm{r}} = 0 \tag{17}$$

$$B_{\mathrm{n}}n_{\mathrm{i}} - B_{\mathrm{p}}p_{\mathrm{i}} - A_{\mathrm{n}}n_{\mathrm{r}} - A_{\mathrm{p}}p_{\mathrm{r}} = -G_{\mathrm{ac}} \tag{18}$$

$$A^*_{\mathrm{n}}n_{\mathrm{i}} + A^*_{\mathrm{p}}p_{\mathrm{i}} + B^*_{\mathrm{n}}n_{\mathrm{r}} - B^*_{\mathrm{p}}p_{\mathrm{r}} = 0 \tag{19}$$

$$-B^*_{\mathrm{n}}n_{\mathrm{i}} + B^*_{\mathrm{p}}p_{\mathrm{i}} - A^*_{\mathrm{n}}n_{\mathrm{r}} - A^*_{\mathrm{p}}p_{\mathrm{r}} = -G_{\mathrm{ac}}. \tag{20}$$

In the further course of their derivation, Longeaud et al. show that this system of equation can be solved by simplifying and to some extent also neglecting the coefficients A_{n}, A_{p}, B_{n}, B_{p}, $A^{\star}_{\mathrm{n}}$, $A^{\star}_{\mathrm{p}}$, $B^{\star}_{\mathrm{n}}$ and $B^{\star}_{\mathrm{p}}$. However, these coefficients change upon

introducing the additional energy dependencies from above, which leads to the question whether they still can be treated as in the original derivation.

The original expressions for the coefficients A_n, A_p, B_n, B_p, $A^\star_\mathrm{n}$, $A^\star_\mathrm{p}$, $B^\star_\mathrm{n}$ and $B^\star_\mathrm{p}$, which can be found for example in[4], comprise integrals with respect to energy. When evaluating these integrals, it is crucial to consider the energy dependence of the altered capture coefficient $c_{\mathrm{n/p}}(E)$. The revised coefficients read (including $1/\tau = \bar{n}(E)+\bar{p}(E)+e_\mathrm{n}(E)+e_\mathrm{p}(E)$ and $f_\mathrm{dc}(E) = (\bar{n}_\mathrm{dc}(E)+e_\mathrm{p}(E))/(\bar{n}_\mathrm{dc}(E)+\bar{p}_\mathrm{dc}(E)+e_\mathrm{n}(E)+e_\mathrm{p}(E))$):

$$A_\mathrm{n} = \int_{E_\mathrm{V}}^{E_\mathrm{C}} c_\mathrm{n}(E)\cdot\left[1-\frac{\bar{n}_\mathrm{dc}(E)+e_\mathrm{n}(E)}{1/\tau(E)+\tau(E)\omega^2}\right][1-f_\mathrm{dc}(E)]N(E)\mathrm{d}E \tag{21}$$

$$\begin{aligned}
A_\mathrm{p} &= \int_{E_\mathrm{V}}^{E_\mathrm{C}} c_\mathrm{p}(E)\cdot\left[\frac{\bar{n}_\mathrm{dc}(E)+e_\mathrm{n}(E)}{1/\tau(E)+\tau(E)\omega^2}\right] f_\mathrm{dc}(E)N(E)\mathrm{d}E \\
B_\mathrm{n} &= \omega\cdot\int_{E_\mathrm{V}}^{E_\mathrm{C}} c_\mathrm{n}(E)\cdot\left[\frac{\bar{n}_\mathrm{dc}(E)+e_\mathrm{n}(E)}{1/\tau^2(E)+\omega^2}\right][1-f_\mathrm{dc}(E)]N(E)\mathrm{d}E+\omega\cdot c_\mathrm{n}(E) \\
B_\mathrm{p} &= \omega\cdot\int_{E_\mathrm{V}}^{E_\mathrm{C}} c_\mathrm{p}(E)\cdot\left[\frac{\bar{n}_\mathrm{dc}(E)+e_\mathrm{n}(E)}{1/\tau^2(E)+\omega^2}\right] f_\mathrm{dc}(E)N(E)\mathrm{d}E \\
A^\star_\mathrm{n} &= \int_{E_\mathrm{V}}^{E_\mathrm{C}} c_\mathrm{n}(E)\cdot\left[1-\frac{\bar{p}_\mathrm{dc}(E)+e_\mathrm{p}(E)}{1/\tau(E)+\tau(E)\omega^2}\right][1-f_\mathrm{dc}(E)]N(E)\mathrm{d}E \\
A^\star_\mathrm{p} &= \int_{E_\mathrm{V}}^{E_\mathrm{C}} c_\mathrm{p}(E)\cdot\left[1-\frac{\bar{p}_\mathrm{dc}(E)+e_\mathrm{p}(E)}{1/\tau(E)+\tau(E)\omega^2}\right] f_\mathrm{dc}(E)N(E)\mathrm{d}E \\
B^\star_\mathrm{n} &= \omega\cdot\int_{E_\mathrm{V}}^{E_\mathrm{C}} c_\mathrm{n}(E)\cdot\left[\frac{\bar{p}_\mathrm{dc}(E)+e_\mathrm{p}(E)}{1/\tau^2(E)+\omega^2}\right][1-f_\mathrm{dc}(E)]N(E)\mathrm{d}E \\
B^\star_\mathrm{p} &= \omega\cdot\int_{E_\mathrm{V}}^{E_\mathrm{C}} c_\mathrm{p}(E)\cdot\left[\frac{\bar{p}_\mathrm{dc}(E)+e_\mathrm{p}(E)}{1/\tau^2(E)+\omega^2}\right] f_\mathrm{dc}(E)N(E)\mathrm{d}E+\omega\cdot c_\mathrm{n}(E)\,.
\end{aligned} \tag{22}$$

As it is described in[20], it is useful to identify the expressions χ, $\chi^\star$, χ/τ and $\chi^\star\tau$ in the original set of coefficients. The same approach can be applied to the revised coefficients above, provided that the expressions for χ, $\chi^\star$, χ/τ and $\chi^\star/\tau$ from[20] are modified:

$$\begin{aligned}
\chi &= c_\mathrm{n}(E)\cdot\left[\frac{\bar{n}_\mathrm{dc}(E)+e_\mathrm{n}(E)}{1/\tau^2(E)+\omega^2}\right] \\
\chi^\star &= c_\mathrm{p}(E)\cdot\left[\frac{\bar{p}_\mathrm{dc}(E)+e_\mathrm{p}(E)}{1/\tau^2(E)+\omega^2}\right] \\
\chi/\tau &= c_\mathrm{n}(E)\cdot\left[\frac{\bar{n}_\mathrm{dc}(E)+e_\mathrm{n}(E)}{1/\tau(E)+\tau(E)\omega^2}\right] \\
\chi^\star/\tau &= c_\mathrm{p}(E)\cdot\left[\frac{\bar{p}_\mathrm{dc}(E)+e_\mathrm{p}(E)}{1/\tau(E)+\tau(E)\omega^2}\right]\,.
\end{aligned} \tag{23}$$

Considering these modified expression, large parts of the integrands in equations 22 can be written by means of χ, $\chi^\star$, χ/τ, $\chi^\star/\tau$ and f_dc. Thus, finding approximations for these five quantities also serve the purpose of approximating the coefficients from equations 22. As reasoned by Longeaud[4], the integrals with respect to energy across the entire bandgap from E_V to E_C are split into five integrals over five respective energy regions. Essentially, this step results from dividing the bandgap into the recombination zone between the quasi Fermi levels for trapped electrons and holes ($E^\mathrm{p}_\mathrm{Ft} \leq E \leq E^\mathrm{n}_\mathrm{Ft}$) and the two remaining energy regions between the bandedges and the quasi Fermi levels. Each of the latter ones is again divided into two sub regions due to the energy level at which the DOS is probed by means of electrons/holes ($E_{\omega\mathrm{n}}/E_{\omega\mathrm{p}}$). In the original analysis of Longeaud, these pronounced energy levels relate to the energy levels at which the emission rate of states equals the modulation frequency ($e_\mathrm{p}(E_{\omega\mathrm{p}}) = e_\mathrm{p}(E_{\omega\mathrm{p}}) = \omega$). At this point, we anticipate the existence of such a pronounced energy also for the modified MPC analysis considering multiphonon transitions. This assumption is justified as it can be seen below, even though the relation between modulation frequency and emission rate comprises an additional multiplication factor (see equation 30).

Approximations for the expressions of χ, $\chi^\star$, χ/τ, $\chi^\star/\tau$ and f_dc are given in table 1 and according illustrations can be found in figure 8 based on typical DOS parameters for amorphous PCM. As it can be seen, the contribution of χ, $\chi^\star$, χ/τ and $\chi^\star/\tau$ to the coefficients from equations 22 can be neglected for large parts of the bandgap. This results in major simplifications of the integrals in equations 22, as it will be discussed to the example of $B_{\mathrm{n/p}}$ further below. Apart from this, attention is drawn to the occupation function f_dc as illustrated in the lower part of figure 8. When not considering multiphonon transition probabilities, the occupation function exhibits the well-known two-step characteristic[2,21]. Therein, the recombination zone is

located around the bandgap center and comprises states for which the emission rate of both electrons and holes is far below the respective trapping rates. The latter two are assumed to be independent of energy when neglecting multiphonon transitions, which leads to the occupation function being constant within the recombination zone. However, upon taking account of multiphonon transitions and thus introducing trapping rates that depend exponentially on energy ($\bar{n}(E)$ and $\bar{p}(E)$ as mentioned above), the occupation function in the recombination zone features a non-constant occupation probability. This phenomenon was observed in a similar manner by Taylor and Simmons studying the effect of energy-dependent capture cross sections on the occupation function[2]. In view of the task at hand to simplify the coefficients from equations 22, it can be stated that the revised, more complex occupation function can still be approximated reasonably within the recombination zone.

energy range	χ	$\chi^\star$	χ/τ	$\chi^\star/\tau$	f_{dc}
$E_\mathrm{V} \leq E \leq E_{\omega\mathrm{p}}$	0	$c_\mathrm{p}(E)\cdot\dfrac{e_\mathrm{p}(E)}{e_\mathrm{p}(E)^2+\omega^2}$	0	$c_\mathrm{p}(E)$	1
$E_{\omega\mathrm{p}} \leq E \leq E_\mathrm{Ft}^\mathrm{p}$	0	$c_\mathrm{p}(E)\cdot\dfrac{e_\mathrm{p}(E)}{e_\mathrm{p}(E)^2+\omega^2}$	0	0	1
$E_\mathrm{Ft}^\mathrm{p} \leq E \leq E_\mathrm{Ft}^\mathrm{n}$	0	0	0	0	$\dfrac{\bar{n}_\mathrm{dc}(E)}{\bar{n}_\mathrm{dc}(E)+\bar{p}_\mathrm{dc}(E)}$
$E_\mathrm{Ft}^\mathrm{n} \leq E \leq E_{\omega\mathrm{n}}$	$c_\mathrm{n}(E)\cdot\dfrac{e_\mathrm{n}(E)}{e_\mathrm{n}(E)^2+\omega^2}$	0	0	0	0
$E_{\omega\mathrm{n}} \leq E \leq E_\mathrm{C}$	$c_\mathrm{n}(E)\cdot\dfrac{e_\mathrm{n}(E)}{e_\mathrm{n}(E)^2+\omega^2}$	$c_\mathrm{n}(E)$	0	0	0

Table 1. Approximating functions for the expressions of χ, $\chi^\star$, χ/τ, $\chi^\star/\tau$ and f_{dc}. The integrands in equations 22 can be significantly simplified by identifying the expression of χ, $\chi^\star$, χ/τ, $\chi^\star/\tau$ (see equations 23) and approximating these expressions. As it turns out, the contribution of these expressions and the occupation function f_{dc} to the coefficients of equations 22 can be neglected for large parts of the bandgap. An illustration of the given approximations can be found in figure 8.

As it results from the approximations in table 1, the dominant coefficients in the linear system of equations 20 are A_n, B_n, $A_\mathrm{p}^\star$ and $B_\mathrm{p}^\star$. Following the original derivation steps of Longeaud[4], the coefficients $B_\mathrm{n}/B_\mathrm{p}^\star$ play a crucial role when eventually linking the DOS and experimental MPC data. Considering that holes are the dominating charge carrier type in amorphous PCM and further assuming hat probed localised states are located far away from the recombination zone, the following equation can be developed:

$$|I_\mathrm{ac}| \approx \frac{G_\mathrm{ac} S q \xi \mu_\mathrm{ext,p}}{\sqrt{(B_\mathrm{p}^\star)^2/\tan(\phi)+(B_\mathrm{p}^\star)^2}}\,, \tag{24}$$

including the amplitude $|I_\mathrm{ac}|$ and phase ϕ of the modulated photocurrent, the modulated component of the photogeneration rate G_ac, the current cross section S, the elementary charge q, the applied electrical field ξ and the extended-state mobility of holes $\mu_\mathrm{ext,p}$. By neglecting multiphonon transitions, $B_\mathrm{p}^\star$ in equation 24 was originally given by:

$$B_\mathrm{p}^\star = \omega + c_\mathrm{p}\cdot\int_{E_\mathrm{V}}^{E_\mathrm{Ft}^\mathrm{p}} \underbrace{\frac{\omega\cdot e_\mathrm{p}(E)}{e_\mathrm{p}^2(E)+\omega^2}}_{G_\mathrm{p}(E)} N(E)\mathrm{d}E\,. \tag{25}$$

For the interpretation of equation 25, it is useful to briefly recall the underlying principle of the original MPC analysis[4]. Therein, it is assumed that the MPC signal is dominated by charge carriers released from states whose emission rate is equal to the modulation frequency. This basic assumption goes back to the sharp peak that is found in the traditional weighting function $G_\mathrm{p}(E)$ in equation 25, which is centred at the energy level satisfying the condition of $e_\mathrm{p}(E_{\omega\mathrm{p}}) = \omega$. Longeaud et al. made use of the sharp peak form of $G_\mathrm{p}(E)$ at the probed energy level $E_{\omega\mathrm{p}}$ and solved the integral in equation 25 by approximating $G_\mathrm{p}(E)$ by a Dirac delta function. To this end, a normalisation factor needs to be calculated by integrating the weighting function with respect to energy from $-\infty$ to $+\infty$. Deriving this normalisation factor for the original weighting function is straightforward and

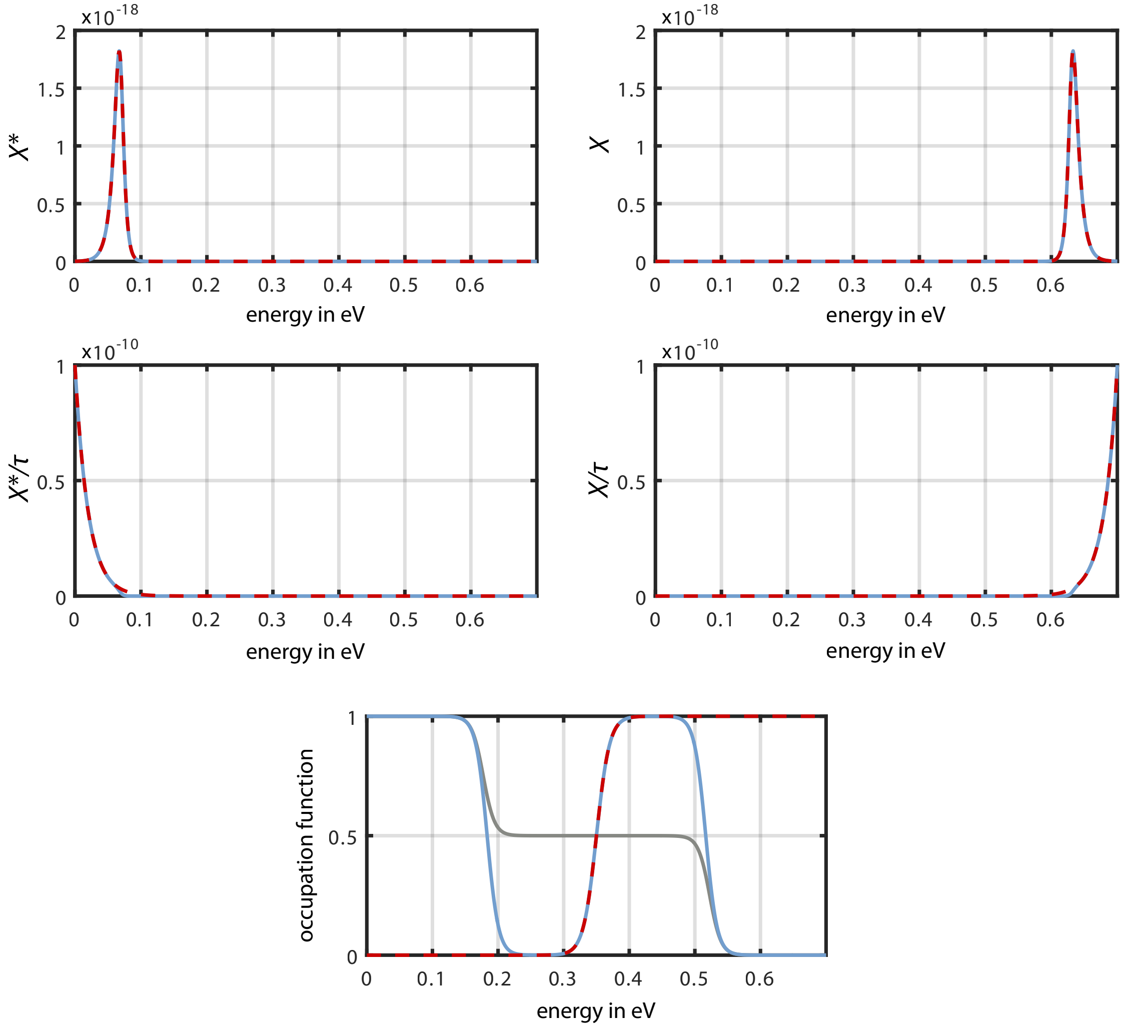

Figure 8. Illustration of the expressions for χ, $\chi^{\star}$, χ/τ, $\chi^{\star}/\tau$ and f_{dc} and corresponding approximations. The approximating functions given in table 1 are drawn in dashed red lines and the full expressions for χ, $\chi^{\star}$, χ/τ, $\chi^{\star}/\tau$ and f_{dc} (see also equations 23) are drawn in full blue lines. Upon considering multiphonon transitions, the occupation function deviates from the well-known two-step function based on assuming constant capture coefficients (grey line). Typical DOS parameters for amorphous PCM are assumed: $N_{\mathrm{V}}(E_{\mathrm{V}}) = N_{\mathrm{C}}(E_{\mathrm{G}}) = 10^{23}\,\mathrm{cm}^{-3}\mathrm{eV}^{-1}$, $T_{\mathrm{V}} = 100\,\mathrm{K}$, $E_{\mathrm{G}} = 0.7\,\mathrm{eV}$, $\sigma_{\mathrm{n/p}} \cdot \upsilon_{\mathrm{th}} = 1 \cdot 10^{-10}\,\mathrm{cm}^3\mathrm{s}^{-1}$, $\hbar\omega_0 = 0.02\,\mathrm{eV}$. This yields $E_{\omega\mathrm{p}} = 0.067\,\mathrm{eV}$, $E^{\mathrm{p}}_{\mathrm{Ft}} = 0.183\,\mathrm{eV}$, $E^{\mathrm{n}}_{\mathrm{Ft}} = 0.517\,\mathrm{eV}$, $E_{\omega\mathrm{n}} = 0.633\,\mathrm{eV}$.

yields $(\pi/2)\cdot k_{\rm B}T$, which leads to:

$$\begin{aligned} B_{\rm p}^{\star} &= \omega + c_{\rm p}k_{\rm B}T\frac{\pi}{2}\cdot\int_{E_{\rm V}}^{E_{\rm Ft}^{\rm p}}\delta(E_{\omega{\rm p}}-E)N(E){\rm d}E \\ &= \omega + c_{\rm p}k_{\rm B}T\frac{\pi}{2}N(E_{\omega{\rm p}})\,. \end{aligned} \tag{26}$$

Eventually, combining the outcome of equation 26 and equation 24 within the original framework leads to

$$N(E_{\omega{\rm p}}) = \frac{2}{\pi c_{\rm p}k_{\rm B}T}\cdot\left[\frac{\mu_{\rm ext,p}q\xi SG_{\rm ac}\sin(\phi)}{|I_{\rm ac}|}-\omega\right]. \tag{27}$$

Since the modulation frequency ω is generally negligible compared to the first term within the large parantheses of equation 27, an analytical expression linking the DOS $N(E)$ and experimental MPC data (amplitude $|I_{\rm ac}|(\omega)$ and phase $\phi(\omega)$ of the modulated photocurrent) is obtained (also given in equation (1) of the main text):

$$\frac{N(E_{\omega{\rm p}})\cdot c_{\rm p}}{\mu_{\rm ext,p}} = \frac{2}{\pi k_{\rm B}T}\cdot\frac{q\xi SG_{\rm ac}\sin(\phi)}{|I_{\rm ac}|}\,. \tag{28}$$

In the present case of considering the multiphonon interpretation of charge carrier transitions, the coefficient $B_{\rm p}^{\star}$ fed into equation 24 is more complex. That is because it includes the energy-dependent capture coefficient $c_{\rm p}(E)$ in contrast to the traditional expression for $B_{\rm p}^{\star}$ (equation 25). Taken into account $c_{\rm p}(E)$ when solving the integral with respect to energy leads to:

$$B_{\rm p}^{\star} = \omega + \int_{E_{\rm V}}^{E_{\rm Ft}^{\rm p}}\underbrace{c_{\rm p}(E)\cdot\frac{\omega\cdot e_{\rm p}(E)}{e_{\rm p}^2(E)+\omega^2}}_{G'_{\rm p}(E)}N(E){\rm d}E\,. \tag{29}$$

In this alternative expression for $B_{\rm p}^{\star}$, the modified weighting function $G'_{\rm p}(E)$ can be identified. Compared to the original weighting function, $G'_{\rm p}(E)$ additionally comprises the energy-dependent capture coefficient $c_{\rm p}(E)$. Still, this revised weighting function exhibits a sharp peak as illustrated in the left panel of figure 9. Calculating the first derivative reveals that at the peak it holds

$$\omega = \frac{1}{\sqrt{\frac{2k_{\rm B}T}{\hbar\omega_0}+1}}\cdot\underbrace{N_{\rm V,eff}\cdot\sigma_{\rm p}\cdot\upsilon_{\rm th}\cdot\exp\left(-[E-E_{\rm V}]/\hbar\omega_0\right)\cdot\exp\left(-[E-E_{\rm V}]/k_{\rm B}T\right)}_{e_{\rm p}(E)}\,. \tag{30}$$

Mathematically speaking, the original condition of $e_{\rm p}(E_{\omega{\rm p}})=\omega$ is simply complemented by an additional multiplication factor $1/\sqrt{2k_{\rm B}T/\hbar\omega_0+1}$. Thus, the energetic position of the peak still is a function of the modulation frequency ω, which means that energy levels still can be selectively probed by setting ω. Additionally, because of said peak in the revised weighting function, approximating $G'_{\rm p}(E)$ by a Dirac delta function might still be a feasible approach for solving the expression for $B_{\rm p}^{\star}$ in equation 29. However, difficulties may arise from the asymmetric shape of the peak in $G'_{\rm p}(E)$ (right panel of figure 9) and also from the fact that calculating the normalization factor via integration of $G'_{\rm p}(E)$ with respect to energy from $-\infty$ to $+\infty$ appears to be less straightforward. Numerical calculations reveal that compared to the original normalisation factor, the revised normalisation factor additionally depends on the modulation frequency and comprises a rather complex dependence on temperature. This implies that if an analytical expression for $B_{\rm p}^{\star}$ can be found and used as input for equation 24, the resulting link between DOS and experimental MPC signal may be more complex compared to the traditional analysis (equation 28).

It can be summarised that under the consideration of multiphonon transitions, the revised weighting function still exhibits a sharp peak whose energetic position can be shifted by varying ω. This means that the fundamental MPC spectroscopy principle of probing localised states at a pronounced energy level by setting a specific modulation frequency remains valid. Nevertheless, establishing an analytical link between the DOS and the experimental MPC data appears to be less straightforward due to the additional energy dependence of the capture coefficient. Eventually, it might turn out to be more feasible to apply numerical methods as part of the revised MPC analysis. Such a revised MPC analysis might allow the extraction of quantitative results on the DOS and the capture coefficient, while taking multiphonon transitions into account. The approach used in the present work, however, is limited to qualitative conclusions.

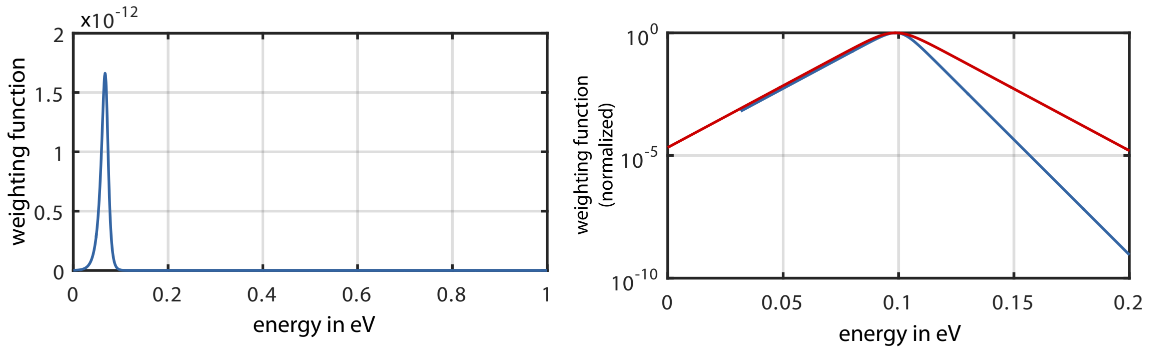

Figure 9. Pronounced peak in the revised weighting function. Left: Similar to the original weighting function $G_p(E)$, the revised weighting function $G'_p(E)$ drawn as blue line exhibits a sharp peak. **Right:** Comparing original (red line) and revised (blue line) weighting function reveals the asymmetric peak of the revised weighting function. The same DOS parameters as for the illustration in figure 8 are used. To facilitate the comparison, the peaks of both weighting functions are normalized and shifted to a common energy value.

References

1. Kleider, J.-P., Longeaud, C. & Gueunier, M.-E. The modulated photocurrent technique: a powerful tool to investigate band gap states in silicon based thin films. *physica status solidi (c)* **1**, 1208–1226 (2004). DOI 10.1002/pssc.200304322.
2. Simmons, J. & Taylor, G. Nonequilibrium steady-state statistics and associated effects for insulators and semiconductors containing an arbitrary distribution of traps. *Phys. Rev. B* **4**, 502–511 (1971). DOI 10.1103/PhysRevB.4.502.
3. Shockley, W. & Read, W. Statistics of the recombinations of holes and electrons. *Phys. Rev.* **87**, 835–842 (1952). DOI 10.1103/PhysRev.87.835.
4. Longeaud, C. & Kleider, J. General analysis of the modulated-photocurrent experiment including the contributions of holes and electrons. *Phys. Rev. B* **45**, 11672–11684 (1992). DOI 10.1103/PhysRevB.45.11672.
5. Varshni, Y. P. Temperature dependence of the energy gap in semiconductors. *Phys.* **34**, 149–154 (1967). DOI 10.1016/0031-8914(67)90062-6.
6. Kaes, M. & Salinga, M. Impact of defect occupation on conduction in amorphous $Ge_2Sb_2Te_5$. *Sci. reports* **6**, 31699 (2016). DOI 10.1038/srep31699.
7. Luckas, J. *et al.* The influence of a temperature dependent bandgap on the energy scale of modulated photocurrent experiments. *J. Appl. Phys.* **110**, 013719 (2011). DOI 10.1063/1.3605517.
8. Fan, H. Y. Temperature dependence of the energy gap in semiconductors. *Phys. Rev.* **82**, 900–905 (1951). DOI 10.1103/PhysRev.82.900.
9. Fan, H. Y. Temperature dependence of the energy gap in monatomic semiconductors. *Phys. Rev.* **78**, 808–809 (1950). DOI 10.1103/PhysRev.78.808.2.
10. Spear, W. E., Allan, D., Le Comber, P. & Ghaith, A. A new approach to the interpretation of transport results in a-Si. *Philos. Mag. Part B* **41**, 419–438 (1980). DOI 10.1080/13642818008245397.
11. Spear, W. E., Al-Ani, H. & Le Comber, P. G. Photoconductivity studies of the mobility edge in amorphous silicon. *Philos. Mag. Part B* **43**, 781–796 (1981). DOI 10.1080/01418638108222346.
12. Rütten, M., Kaes, M., Albert, A., Wuttig, M. & Salinga, M. Relation between bandgap and resistance drift in amorphous phase change materials. *Sci. reports* **5**, 17362 (2015). DOI 10.1038/srep17362.
13. Longeaud, C. & Tobbeche, S. The influence of hopping on modulated photoconductivity. *J. physics. Condens. matter : an Inst. Phys. journal* **21**, 045508 (2009). DOI 10.1088/0953-8984/21/4/045508.
14. Krebs, D., Bachmann, T., Jonnalagadda, P., Dellmann, L. & Raoux, S. Changes in electrical transport and density of states of phase change materials upon resistance drift. *New J. Phys.* **16**, 043015 (2014). DOI 10.1088/1367-2630/16/4/043015.
15. Marshall, J. M. Analytical procedures for the modelling of hopping transport in disordered semiconductors. *Philos. Mag. Lett.* **80**, 691–701 (2000). DOI 10.1080/09500830050143796.

16. Merazga, A., Tobbeche, S., Main, C., Al-Shahrani, A. & Reynolds, S. Numerical simulation of the steady state photoconductivity in hydrogenated amorphous silicon including localized state electron hopping. *J. Physics: Condens. Matter* **18**, 3721–3734 (2006). DOI 10.1088/0953-8984/18/15/017.

17. Galperin, Y., Karpov, V. & Kozub, V. Localized states in glasses. *Adv. Phys.* **38**, 669–737 (1989). DOI 10.1080/00018738900101162.

18. Baranovskii, S., Karpov, V. & Shklovskii, B. Nonradiative recombination in noncrystalline semiconductors. *Zh. Éksp. Teor. Fiz* **94**, 288 (1988).

19. Baranovskii, S. & Karpov, V. Localized electron states in glassy semiconductors. *Sov. Phys. - Semicond. Transl.* **21**, 1–10 (1987).

20. Luckas, J. *Investigating defect states in phase-change materials using modulated photo current experiments*. Master thesis, RWTH Aachen University (2008).

21. Taylor, G. & Simmons, J. Basic equations for statistics, recombination processes, and photoconductivity in amorphous insulators and semiconductors. *J. Non-Crystalline Solids* **8-10**, 940–946 (1972). DOI 10.1016/0022-3093(72)90250-5.
